# Supplementary material for: Evidence for the effectiveness of interventions to reduce mental health related stigma in the workplace: a systematic review
Source: BMJ Open. 2023 Feb 20;13(2):e067126. doi: 10.1136/bmjopen-2022-067126 (PMC9944311; doi:10.1136/bmjopen-2022-067126)
Supplement: Supplementary data [file bmjopen-2022-067126supp001.pdf]

Appendix 1:

The following TI/AB keywords were used:

depress* OR suic* OR anx* OR self-harm OR "mental health" OR discrimination OR exclusion
AND
occupation* or workplace or SME OR job OR "small-sized enterprise*" OR "medium-sized enterprise*" OR "small enterprise*" OR "medium enterprise*" OR "small-sized compan*" OR "medium-sized compan*" OR "small compan*" OR "medium compan*" OR "small-sized business*" OR "medium-sized business*" OR "small business*" OR "medium business*" OR "small-sized organization*" OR "small-sized organisation*" OR "medium-sized organization*" OR "medium-sized organisation*" OR "small organization*" OR "small organisation*" OR "medium organization*" OR "medium organisation*")
AND
anti-stigma OR stigma
AND
reduced OR promot* OR program* OR campaign OR improve* OR intervention OR educat* OR seminar* OR workshop* OR course
