# Supplementary material for: Evidence for the effectiveness of interventions to reduce mental health related stigma in the workplace: a systematic review
Source: BMJ Open. 2023 Feb 20;13(2):e067126. doi: 10.1136/bmjopen-2022-067126 (PMC9944311; doi:10.1136/bmjopen-2022-067126)
Supplement: Supplementary data [file bmjopen-2022-067126supp002.pdf]

Supplementary table 1. Overview of study characteristics

First author/ year	Study Design	Population at baseline and follow up	Gender at baseline	Target group	Sector/ Size of organization	Intervention	Intervention Intensity	Country
**Bond et al, 2021**	longitudinal cohort study	284 pre<br>98 post	212 female<br>72 male	support services, police, educators and general community networks	public sector	Mental Health First Aid for the Suicidal Person course	4-hours course	Australia
**Dimoff 2016**	controlled study active vs wait list	183 pre<br>142 post	77 female<br>65 male	Leaders in telecommunication companies	large company, private sector	Mental health awareness training	3 hours training	Canada
**Dobson et al, 2021**	cluster-randomized trial	123 pre<br>101 post	115 female<br>8 male	office workers<br><br>kitchen and maintenance staff	large company, public sector	The Working Mind program:<br>• trained facilitators,<br>• workshop manuals,<br>• contact-based videos<br>• discussion exercises,<br>• personal goal setting.<br>Participants allocated to immadiate or delayed intervention groups.	4-hours group program	Canada
**Dobson et al.,<br><br>2019**	open trial methodology	1292 pre<br>1155 post	male 419<br>female 719	government, education, health, energy supervisors and frontline staff	public sector	The Working Mind Program<br>• trained facilitators,<br>• workshop manuals,<br>• contact-based videos<br>• discussion exercises,<br>• personal goal setting.<br>“train-the- trainer” model	Two versions:<br>4-hour group program for frontline workers<br><br>8-hour program for managers	Canada
**Eiroa-Orosa et al, 2021**	cluster randomized-	371 pre<br>260 post	314 female<br>57 male	primary health and mental health care professionals	public sector	awareness-raising intervention<br><br>The Targeted, Local, Credible, Continuous Contact (TLC3)	**4 workshops**<br>1. Training: pedagogy and contact (face-to face +video) – 4 hours	Spain

	controlled trial			administrative officers, general practitioners, odontologists, nurses, psychiatrists, psycholhologists, and social workers.		methodology adapted to the Catalan healthcare context	2. self-diagnosis and prioritization – 4 hours<br>3. self-organized activities<br>4. follow-up session	
**Griffith et al 2016**	Randomised controlled trial	507 pre<br>386 post	MH-guru: male: 29%, female: 70%;	multi-departmental government workplace	public sector	online depression and anxiety educational workplace induction program ("Mental Health Guru"): two modules: depression and General anxiety disorder	1 hour long online depression and anxiety educational program (1 module/week, 30min/module)	Australia
**Hamann et al., 2016**	Longitudinal cohort study	580 pre	210 women<br>370 men	Leaders, members of the workers' council, workers in HR department	not specified companies (n=30)	"Mental-health-at-the-workplace" educational workshop	1-1,5 days training	Germany
**Hanisch et al., 2017**	Longitudinal cohort study	48 pre	92% male, 8% female	Leaders	private sector – large enterprise	"Leadership Training in Mental Health Promotion" (LMHP), a digital game-based training program for leaders which is combining games and simulations in a virtual environment.	1.5- 2 hours long single session	UK
**Kristman et al., 2019**	quasi-experimental	89 pre<br>61 post	59 male<br>24 female	Leaders in HR, occupational health and safety management	public and private sector, different size companies	Multi-faceted:<br>1. "Standard to Action" training program designed to help employers implement the Standard in	2 years	Canada

						their workplaces – 6 sessions;<br>2. Education sessions: MH First Aid sessions;<br>3. Social marketing campaign including a photovoice exhibit.		
**Kubo et al, 2018**	Single arm pilot trial	91 pre<br>83 post	male 77%<br>female 23%	office workers	no specific information	"Mental Health First Aid" (MHFA) training program modified for workplace settings.	2-hour training course	Japan
**Moffitt et al, 2014**	random allocation design	106 pre<br>89 post	N/A	fire service line managers	public sector	Participants randomly assigned to:<br>• Looking after Wellbeing at Work" (LWW)<br>• Mental Health First Aid (MHFA)<br>• leaflet session (LS).	LWW- 2days<br>MHFA – 12 hours<br>LS- 1 hour	UK
**Moll et al, 2018**	randomised, parallel-group trial	192 pre<br>167 post<br>150 by 6 mo follow up	female 88.5%<br>male 11.5%	Healthcare workers	public sector	"Beyond Silence" (Beyond Silence program includes a contact-based educational approach)<br>Mental Health First Aid training	comprising 6 in-person, 2-h sessions + 5 online sessions co-led by employees who personally experienced mental health issues<br>standardised 2-days training program	Canada

**Paterson et al, 2021**		134 pre<br>57 post	not specified	not specified	public and private, different size companies	Headtorch WORKS - mental health and well-being intervention	3 online episodes + discussion group<br>6 hours<br>original filmed drama and specialist documentary	UK
**Quinn et al, 2011**		101 pre<br>87 post	77% male<br>23% female	housing association and telecommunication workers	public and private sector	Training course on mental health awareness	One day training (6 hours each)<br><br>combination of service user narratives, experiential group learning, and didactic teaching approaches.	Scotland
**Reavley et al., 2018**	Randomized controlled trial	608 pre<br>289 post	449 female<br>159 male	public servants	public sector	Participants randomized to<br><br>-eLearning MHFA,<br><br>-blended MHFA<br><br>-PFA eLearning	- 6-hour eLearning MHFA online course<br>- 6-hour eLearning MHFA plus 4-hour face-to-face session<br>- 4-hour eLearning PFA online course	Australia
**Shann et al., 2018**	Randomized controlled trial	311 pre<br>196 post	148 male<br>163 female	Leaders	public<br>private<br>non- profit<br>other sectors 1%	"Beyondblue" online materials for leaders:<br>Main focus on depression:<br>-written information,<br>-video clips of organizational leaders speaking<br>about mental health in the workplace,<br>-interactive exercises in which participants can calculate the cost of untreated depression and the	30 – 45 min	Australia

						specific risk factors in their organization.		
**Svensson and Hansson, 2014**	Randomized controlled trial	416 pre<br>277 post	151 female<br>48 male	Not specified employees	public sector	Mental Health First Aid training	12 hours course,<br>spread over two days	Sweden
**Szeto et al. 2019**	non-randomized quasi-experimental	5598 pre<br>4649 post<br><br>Frontline staff 75.8% (3,449)<br>Supervisory staff 26.4% (1,210)	male 55.9%<br>female 44.1%	Corrections 9.0% (418)<br>Emergency Services (9-1-1) 3.9% (192)<br>Fire Services 17.7% (821)<br>Police Services 56.5% (2,623)<br>Paramedics 13.0% (605)	public sector	"Road to Mental Readiness for First Responders" program (R2MR)<br>**3 main components**:<br>stigma reduction through video contact-based education,<br>the Mental Health Continuum Model, and<br>"Big 4" coping and resilience skills.<br>Additional skills for supervisors.	4-hour program for employees<br><br>8-hour program for supervisors	Canada
**Tynan et al. 2018**	Non-Randomised controlled trial	1275 pre<br>1163 post<br><br>Supervisor:<br>117 pre<br>114 post	1014 male;<br>135 female;<br><br>Supervisor training:<br>92 male;<br>10 female;<br>12 not specifed.	Manager<br>Professional<br>Trades worker<br>Machinery operator Admin or other	private sector, medium and large	"Working Well Mental health Program":<br><br>peer-based, multi-component mental health and suicide prevention program<br><br>supervisor training	- 1 hour 'general awareness training' (GAT),<br>- 4 hours of 'gate-keeper training',<br>-2-day 'Applied Suicide Intervention Skills Training' (ASIST) for key workers.	Australia
