# Supplementary material for: Evidence for the effectiveness of interventions to reduce mental health related stigma in the workplace: a systematic review
Source: BMJ Open. 2023 Feb 20;13(2):e067126. doi: 10.1136/bmjopen-2022-067126 (PMC9944311; doi:10.1136/bmjopen-2022-067126)
Supplement: Supplementary data [file bmjopen-2022-067126supp003.pdf]

Supplementary table 2. Study findings

**Results from Randomized Controlled Trials**				
**First author/year**	**Outcome measure on stigma**	**Evaluation timepoints**	**Main findings**	
Dimoff 2016	Depression Stigma personal Scale (DSS)	pre training<br>post training<br><br>2 months follow up	Significant improvements in stigmatizing attitudes were also observed for the intervention group from T1 (M = 2.98, SD = 0.39) to T2 (M =3.25, SD = 0.37; t (87) = -5.60, p < .001) and from T1 to T3 (M = 3.20, SD = 0.42; t (87) = -4.06, p < .001).<br>No significant improvements in attitude were observed for the intervention group between T2 and T3.	
Dobson et al, 2021	Opening Minds Scale for Workplace Attitudes (OMS-WA)	pre, post training<br>3 months follow up	Stigma scores on the OMS-WA revealed a significant time effect, F(2/154) = 16.33, P < 0.001. There was also a significant group effect, F(1/76) = 16.23, P <0.001, but the interaction effect was not statistically significant, F(2/154) = 1.02, P = 0.362.<br>Pairwise comparison analyses revealed a significant pre- to postreduction in stigma for both the immediate, t(154) = 3.22, P = 0.004, and the delayed group, t(154) = 4.12, P < 0.001.<br>Significant reduction in stigma from pre- to posttest, which was maintained to the time of the follow-up assessment.	
Eiora-Orosa et al, 2021	Opening Minds Scale for Health Care Providers (OMS-HC)<br><br>Beliefs and Attitudes towards Mental Health Service users' rights	pre,<br>1 month follow-up<br>3 months follow up	At baseline statistically significant difference between the intervention and control groups in the total score of the OMS-HC scale (*t* = 2.138, *p* < 0.05)<br><br>Statistically significant decreases were seen between baseline and first follow-up for the OMS-HC total score (*t* = 2.813, *p* < 0.01)<br><br>The general linear models showed a statistically significant drop between the first observation and the second for the OMS-HC disclosure scores with statistically significant effects (*F* = 26.881, *p* < 0.001)<br><br>Reductions in both PC and MH professionals' stigmatising beliefs and attitudes were found in the 1-month follow-up, although a 'rebound effect' at the 3-month follow up was detected.	

Griffith et al. 2016	depression and anxiety personal stigma scale (DSS-personal) (GASS-personal)	baseline,<br>1 week post-intervention<br>6-month follow-up	MH-Guru group showed significantly greater ↓in depression and anxiety personal stigma. Between group effect sizes in stigma for depression were − 0.56 and − 0.47 at post-test and 6-months respectively and − 0.42 at both time points for anxiety (p<.001)<br><br>DSS (Mean, SD)<br>MH-Guru: before: 7.1 (4.9) after: 3.9 (3.8) follow up 4.2 (3.8)<br>Control: Before: 7.3 (5.2) after: 6.8 (5.0) follow up: 6.6 (5.2)<br>F (2, 294.1)=2.5 *P*<.001<br><br>GASS (mean, SD)<br>MH-guru: before: 5.1 (5.1) after: 2.5 (3.9) follow up: 5.1 (0.48)<br>control: before: 4.9 (5.6) after: 5.0 (5.3) follow up: 4.9 (0.34)<br>F (2, 286.1)=19.8 p <.001	
Moffitt, 2014	locally developed "Mental Health Stigma Questionnaire"	pre, post intervention	The LWW and MHFA courses were associated with statistically significant improvements in stigma on mental health.<br>The comparisons showed no significant difference at Time 2 between the LWW and MHFA groups on stigma scale (z= 0.57, p = 0.57, r = 0.07).	
Reavley et al, 2018	Personal Stigma Scale (PSS)	Pre, post training	Those in the blended and eLearning MHFA groups were significantly more likely to show reduced stigma towards people with depression and PTSD than those in the PFA eLearning group.<br>No significant differences between the MHFA eLearning and blended courses.	
Shann et al. 2018.	Managerial Stigma Toward Employee Depression Scale<br>- Affective Stigma Subscale,<br>- Behavioral Stigma Subscale,	pre – post intervention<br>6 month follow up	Significant reductions in behavioral and affective depression-related stigma scores among leaders who completed the intervention, same reduction at 6 months.<br><br>One-way multivariate analysis of covariance showed a statistically significant difference in survey stigma between experimental and control groups, V =.09, F(3, 189)= 6.26, p <.001.<br>Follow-up univariate analyses of variance showed that at posttest, affective stigma was significantly different between groups, F(1, 191) = 14.55, p <.001.	

	- Cognitive Stigma Subscale		The experimental group had lower affective stigma scores (M= 9.42, SEM= .24) at postsurvey compared with the control group (M= 10.51, SEM= .16).	
Svensson and Hansson, 2014	vignette version of the Depression Personal and Perceived Stigma scale (DSS)	pre<br>6 months and<br>2 years follow up	Significant reduction in depression personal stigma after 6 months follow up. Intervention group: pre: 35,8 (5,2) post: 36,3 (4,8). Control group: pre: 36,4 (4.5) post: 35.4 (5.3). F=6,3 p<.05, effect size:0,29.<br><br>The training after two years still have a notable impact on the awareness of mental health and its treatment.	
**Results from quasi-experimental or pre-post design studies**				
Bond et al., 2021	9 statements designed to measure stigmatising attitudes based on Depression Stigma Scale (DSS) – suicide vignette	pre, post training, 6 month follow-up	Reductions in scores on „weak not sick" item after the course and at follow-up (t(275.6)=8.89, p<.0001 and t(132.7)=2.66, p<.0001. Changes in means of „Dangerous/unpredictable"item from pre-course were signifcant both postcourse and at follow-up (t(267.0)=11.74, p<.0001 and t(125.5)=3.81, p=0.0002, respectively).	
Dobson et al.,<br><br>2019	- Stigma towards mental health problems (OMS-WA)	pre program<br>post program<br>3-month follow-up period	The results of the mixed-model analysis revealed statistically significant ↓ in stigma for the total scale, coefficient = .167, SE = .08, z = 20.72, P < 0.001, and all subscales (all Ps < 0.001).<br>The mixed-model analysis for the pre- to post- change on the resiliency skills scale revealed statistically significant improvement at the 95% level of confidence (P < 0.001).	
Hamann et al., 2016	Depression Stigma Scale (DSS)	Pre, post training	significant ↓in personal stigma (mean [SD], 15.5 [3.8]; paired t-test: t = 27.6, p < 0.001)	
Hanisch et al, 2017	-Stigma towards mental health problems (OMS-WA)	pre, post-training, 3-month follow-up	Positive changes on attitudes toward people with mental health problems (*P*<.01).	
Kristman et al., 2019	-Perceived mental health stigma on the workplace	presurvey<br>postsurvey - 2 yrs	Intervention group - pre: mean (SD)= 1.84 (0.74) post: 1.71 (0.64) MD: -0.13, 0.11)	

	Questions derived from Workplace Mental Health in Canada survey.		Significant difference in perceived mental health stigma btw intervention and non-intervention group:<br><br>Intervention group: mean (SD): 1.52 (0.57) vs. Non-intervention group: 2.00 (0.63), MD: -0.48, 95% CI	
Kubo et al. 2018	-Stigma towards mental health problems:<br>Link's Devaluation-Discrimination Scale	pre-program, post-program,<br>1 month follow up	↓ after the program (before: mean (SD)=28.29 (4.9), after: mean (SD) 26.11 (5.36) p=0.003),<br>no difference 1 month after the program. mean (SD): 27.26 (5.78)	
Moll et al.<br>2018	Stigma towards mental health problems – health care (OMS-HC)	presurvey<br>Postsurvey<br>3-mo assessment, 6-mo follow-up	Stigmatized beliefs significantly ↓ in both programs.<br><br>In the stigma analysis, no interactions for treatment arm by time were observed at 3 mo (beta = 0.21, z = 0.22, P = 0.83); although, a possible trend for superior outcomes for Beyond Silence was seen at 6 mo (beta = 1.72, z = 1.7, P = 0.089). To explore whether the anti-stigma effects of Beyond Silence might be more persistent than those of MHFA, a model describing changes from 3 to 6 mo was fit, revealing a significant treatment by time interaction (beta = 1.89, z = 2.09, P 1⁄4 0.037).	
Paterson et al, 2021	Adopted version of King's stigma scale[1]	pre, post intervention	There was no significant difference in the pre/post-intervention change in stigma score between the experimental and control groups.	
Quinn et al, 2011	questions gathered from the Scottish Public Attitudes Survey[2]	pre, post intervention	Attendance at the workshop reduced the level of stigmatizing attitudes for both first (t = 11.939, df = 86, p < 0.0005) and third (t = 3.535, df = 86, p = 0.001) person views. The workshop was associated with a more marked reduction in stigmatizing attitudes	

[1] King, M., Dinos, S., Shaw, J., Watson, R., Stevens, S., Passetti, F., . . . Serfaty, M. (2007). The stigma scale: Development of a standardised measure of the stigma of mental illness. British Journal of Psychiatry, 190(MAR.), 248–254

[2] Braunholtz, S., Davidson, S., & King, S. (2004). Well? What do you think? The second national Scottish survey of public attitudes to mental health, mental well-being and mental health problems. Edinburgh: Scottish Executive.

			expressed by first compared with third person views.	
Szeto et al. 2019	Stigma towards mental health problems (OMS-WA)	pre-program, post-program, 3 month follow up	↓in stigma were observed for the total scale and all subscales. before: 1.97 (SD: 0.47). After: 1.85 (SD: 0.49) coeff: 0.123 SE: 0.008 z: 15.87 p<0.001 Reductions in stigma were maintained until the final follow-up for the total scale. coeff: - 0.002 SE: 0.012 z: - 0.13 p=0.899	
Tynan, 2018	-Mental health stigma, measured by *a perceived stigma* scale[3]	pre-test post-test 10 months follow up	Trend towards a decrease in stigma across both control and intervention sites, however the effect of time or treatment was not signifcant (p > 0.01)	

Key. DSS: Depression Stigma Scale. GASS: The Generalised Anxiety Stigma scale, OMS-WA: Opening Minds Scale for Workplace Attitudes*,* OMS-HC: Opening Minds Scale for Health Care Providers, PSS: Personal Stigma Scale.

[3] Kelly BJ, Stain HJ, Coleman C, Perkins D, Fragar L, Fuller J, Lewin TJ, Lyle D, Carr VJ, Wilson JM, Beard JR. Mental health and well-being within rural communities: the Australian rural mental health study. *Aust J Rural Health*. 2010;18:16–24.
