# Supplementary material for: Evidence for the effectiveness of interventions to reduce mental health related stigma in the workplace: a systematic review
Source: BMJ Open. 2023 Feb 20;13(2):e067126. doi: 10.1136/bmjopen-2022-067126 (PMC9944311; doi:10.1136/bmjopen-2022-067126)
Supplement: Supplementary data [file bmjopen-2022-067126supp004.pdf]

Supplementary table 3: Quality assessment of the selected studies[1]

**Quality of the selected studies**		
WEAK	MODERATE	STRONG
Bond et al, 2021	Dimoff et al, 2016	Moll et al, 2018
Kristman et al, 2019	Dobson et al, 2019	
		Svensson and Hansson, 2014
Kubo et al, 2018	Dobson et al, 2021	
Paterson et al, 2021	Eirosa-Orosa et al, 2021	
Quinn et al, 2011	Griffith et al, 2016	
	Hamann et al, 2016	
	Hanisch et al, 2017	
	Moffitt et al, 2014	
	Reavley et al, 2018	
	Shann et al, 2018	
	Szető et al, 2019	
	Tynan et al, 2018	

1: Based on Quality Assessment Tool for Quantitative Studies (QATQS) scale (Ciliska et al, 1998)
