# Supplementary material for: Evidence for the effectiveness of interventions to reduce mental health related stigma in the workplace: a systematic review
Source: BMJ Open. 2023 Feb 20;13(2):e067126. doi: 10.1136/bmjopen-2022-067126 (PMC9944311; doi:10.1136/bmjopen-2022-067126)
Supplement: Supplementary data [file bmjopen-2022-067126supp005.pdf]

Supplementary Table 4: Quality Assessment of the included studies, based on the QATQS

First Author	Selection Bias	Design	Confounders	Blinding	Data Collection Method	Withdrawals and Drop-out	Global Rating
**Bond et al, 2021**	Strong	Moderate	Weak	Moderate	Strong	Weak	**Weak**
**Dimoff et al, 2016**	Weak	Strong	Strong	Strong	Strong	Moderate	**Moderate**
**Dobson et al, 2019**	Moderate	Moderate	Weak	Moderate	Strong	Strong	**Moderate**
**Dobson et al, 2021**	Moderate	Moderate	Weak	Strong	Strong	Strong	**Moderate**
**Eiroa-Orosa et al, 2021**	Moderate	Strong	Weak	Strong	Strong	Moderate	**Moderate**
**Griffith et al, 2016**	Weak	Strong	Strong	Strong	Strong	Moderate	**Moderate**
**Hamann et al, 2016**	Moderate	Moderate	Weak	Moderate	Strong	Strong	**Moderate**
**Hanisch et al, 2017**	Strong	Moderate	Weak	Moderate	Strong	Strong	**Moderate**
**Kristman et al, 2019**	Weak	Moderate	Weak	Moderate	Weak	Moderate	**Weak**
**Kubo et al, 2018**	Strong	Moderate	Weak	Weak	Strong	Strong	**Weak**
**Moffitt et al, 2014**	Moderate	Strong	Strong	Strong	Weak	Strong	**Moderate**
**Moll et al, 2018**	Moderate	Strong	Strong	Strong	Strong	Moderate	**Strong**
**Paterson et al, 2021**	Weak	Moderate	Weak	Strong	Strong	Weak	**Weak**
**Quinn et al, 2011**	Moderate	Moderate	Weak	Weak	Moderate	Strong	**Weak**
**Reavley et al, 2018**	Strong	Strong	Strong	Strong	Strong	Weak	**Moderate**
**Shann et al, 2018**	Weak	Strong	Strong	Strong	Moderate	Moderate	**Moderate**
**Svensson and Hansson, 2014**	Moderate	Strong	Strong	Strong	Strong	Moderate	**Strong**
**Szető et al, 2019**	Strong	Moderate	Weak	Moderate	Strong	Strong	**Moderate**

**Tynan et al, 2018**	Strong	Moderate	Strong	Strong	Weak	Moderate	**Moderate**
