# Supplementary material for: Evidence for the effectiveness of interventions to reduce mental health related stigma in the workplace: a systematic review
Source: BMJ Open. 2023 Feb 20;13(2):e067126. doi: 10.1136/bmjopen-2022-067126 (PMC9944311; doi:10.1136/bmjopen-2022-067126)
Supplement: Author's manuscript [file bmjopen-2022-067126.draft_revisions.pdf]

BMJ Open is committed to open peer review. As part of this commitment we make the peer review history of every article we publish publicly available.

When an article is published we post the peer reviewers' comments and the authors' responses online. We also post the versions of the paper that were used during peer review. These are the versions that the peer review comments apply to.

The versions of the paper that follow are the versions that were submitted during the peer review process. They are not the versions of record or the final published versions. They should not be cited or distributed as the published version of this manuscript.

BMJ Open is an open access journal and the full, final, typeset and author-corrected version of record of the manuscript is available on our site with no access controls, subscription charges or pay-per-view fees (http://bmjopen.bmj.com).

If you have any questions on BMJ Open's open peer review process please email info.bmjopen@bmj.com

BMJ Open

Evidence for the effectiveness of interventions to reduce mental health related stigma in the workplace: A Systematic Review

Journal:	*BMJ Open*
Manuscript ID	bmjopen-2022-067126
Article Type:	Original research
Date Submitted by the Author:	19-Aug-2022
Complete List of Authors:	Ditta Tóth, Mónika; Semmelweis University, Institute of Behavioural Sciences<br>Ihionvien, Sarah; Semmelweis University, Institute of Behavioural Sciences<br>Leduc, Caleb ; University College Cork, School of Public Health<br>Aust, Birgit; National Research Centre for the Working Environment<br>Amann, Benedikt; Hospital del Mar Institute for Medical Research, Mental Health Group; Univ. Pompeu Fabra<br>Cresswell-Smith, Johanna; THL<br>Reich, Hanna; German Foundation for Research and Education on Depression; German Foundation for Research and Education on Depression<br>Cully, Grace; University College Cork School of Public Health<br>Sanches, Sarita; Phrenos Center of Expertise; Altrecht Foundation for Mental Health Care<br>Fanaj, Naim; Mental Health Center, ; Mental Health Center Prizren<br>Qirjako, Gentiana; University of Medicine, Department of Public Health<br>Tsantila, Fotini; KU Leuven, LUCAS, Centre for Care Research and Consultancy<br>Ross, Victoria ; Australian Institute for Suicide Research and Prevention<br>Mathieu, Sharna; Australian Institute for Suicide Research and Prevention<br>Pashoja, Arlinda; London School of Hygiene and Tropical Medicine Faculty of Public Health and Policy, Faculty of Epidemiology and Population Health<br>Arensman , Ella ; University College Cork, School of Public Health; National Suicide Research Foundation<br>Purebl, György; Semmelweis University, Institute of Behavioural Sciences
Keywords:	MENTAL HEALTH, OCCUPATIONAL & INDUSTRIAL MEDICINE, Depression & mood disorders < PSYCHIATRY

SCHOLARONE™
Manuscripts

I, the Submitting Author has the right to grant and does grant on behalf of all authors of the Work (as defined in the below author licence), an exclusive licence and/or a non-exclusive licence for contributions from authors who are: i) UK Crown employees; ii) where BMJ has agreed a CC-BY licence shall apply, and/or iii) in accordance with the terms applicable for US Federal Government officers or employees acting as part of their official duties; on a worldwide, perpetual, irrevocable, royalty-free basis to BMJ Publishing Group Ltd ("BMJ") its licensees and where the relevant Journal is co-owned by BMJ to the co-owners of the Journal, to publish the Work in this journal and any other BMJ products and to exploit all rights, as set out in our licence.

The Submitting Author accepts and understands that any supply made under these terms is made by BMJ to the Submitting Author unless you are acting as an employee on behalf of your employer or a postgraduate student of an affiliated institution which is paying any applicable article publishing charge ("APC") for Open Access articles. Where the Submitting Author wishes to make the Work available on an Open Access basis (and intends to pay the relevant APC), the terms of reuse of such Open Access shall be governed by a Creative Commons licence – details of these licences and which Creative Commons licence will apply to this Work are set out in our licence referred to above.

Other than as permitted in any relevant BMJ Author's Self Archiving Policies, I confirm this Work has not been accepted for publication elsewhere, is not being considered for publication elsewhere and does not duplicate material already published. I confirm all authors consent to publication of this Work and authorise the granting of this licence.

For peer review only

Evidence for the effectiveness of interventions to reduce mental health related stigma in the workplace: A Systematic Review

Mónika Ditta Tóth[1], Sarah Ihionvien[1], Caleb Leduc[2,3], Birgit Aust[4], Benedikt L. Amann[5,6,7,8,9] Johanna Cresswell-Smith[10], Hanna Reich[11,12], Grace Cully[2], Sarita Sanches[13,14], Naim Fanaj[15], Gentiana Qirjako[16], Fotini Tsantila[17], Victoria Ross[18], Sharna Mathieu[18], Arlinda Cerga Pashoja[19], Ella Arensman[2,3,18], György Purebl[1]

1: Institute of Behavioural Sciences, Semmelweis University, Budapest, Hungary
2: School of Public Health, University College Cork, Cork, Ireland
3: National Suicide Research Foundation, Cork, Ireland
4: National Research Centre for the Working Environment, Copenhagen, Denmark
5: Centre Fòrum Research Unit, Institute of Neuropsychiatry and Addiction (INAD), Parc de Salut Mar, Hospital del Mar Medical Research Institute (IMIM), Barcelona, CIBERSAM, Spain
6: Univ. Pompeu Fabra, Barcelona, Spain
7: Department of Psychiatry and Psychotherapy, Klinikum der Universität München, Munich, Germany
8: Centro de Investigación Biomédica en Red de Salud Mental, Instituto de Salud Carlos III, Madrid, España.
9. Department for Psychiatry and Psychotherapy, Hospital of the Ludwig-Maximilians-University Munich, Germany
[10]Finnish Institute for Health and Welfare (THL)
11: German Depression Foundation, Leipzig, Germany
12: Depression Research Centre of the German Depression Foundation, Department of Psychiatry, Psychosomatic Medicine and Psychotherapy, University Hospital, Goethe University, Frankfurt am Main, Germany
13: Phrenos Center of Expertise for severe mental illness, Utrecht, The Netherlands
14: Altrecht Mental Health Care, Utrecht, The Netherlands
15: Mental Health Center Prizren, Kosovo & College of Medical Sciences Rezonanca Prishtina, Kosovo
16: Department of Public Health, University of Medicine, Tirana, Albania
17: LUCAS, Centre for Care Research and Consultancy, KU Leuven, 3000 Leuven, Belgium;
18: Australian Institute for Suicide Research and Prevention & WHO Collaborating Centre for Research and Training in Suicide Prevention, School of Applied Psychology, Griffith University, Brisbane, Australia
19: Faculty of Epidemiology and Population Health. London School of Hygiene and Tropical Medicine, UK, London

Corresponding author:

Dr. Mónika Ditta Tóth
E-mail: tmonika85@gmail.com
Address: 1089 Budapest, Nagyvárad tér 4. Hungary
Telephone: +36303168621

Keywords:Mental health, stigma, workplace

Word count: 4278

For peer review only

Abstract

Objectives. Increasing access to mental health support is a key factor for treating mental disorders, however, important barriers complicate help-seeking, among them, mental health related stigma being most prominent. Aim. The main aim of this paper is to systematically review the current evidence for interventions focusing on reducing stigma related to mental health problems in Small and Medium Enterprises (SMEs) in various sectors.

Method. A systematic literature search was conducted with a focus on interventions targeting mental health related stigma in the workplace in accordance with PRISMA guidelines. The methodological quality of each included article was assessed using the QATQS scale.

Results. A total of 19 interventions were selected. Only two interventions enrolled SMEs, and no intervention focused on SMEs specifically. Four of the interventions were delivered online, 13 face-to face, while two had a blended design. Our purpose to focus and review interventions with feasible methodology produced positive results. The included interventions produced significant reductions in stigmatizing attitudes for both employees and managers. Positive effects were identified with different size companies, but most of the interventions were evaluated in larger companies. Online interventions are shorter, and can be just as effective as face-to-face interventions.

Conclusions. Although we did not find interventions focusing specifically on SMEs, it is likely that a large proportion of the workforce in these smaller companies may benefit from workplace-based interventions. However, further research with standardized methodology is recommended, with a special focus on their feasibility in smaller enterprises with less resources.

For peer review only

Introduction

Mental disorders can have significant consequences, not only on the individual level, but also on a societal and economic level. In the context of the workplace[1,2], poor mental health has been linked with absenteeism and presenteeism[3, 4, 5] leading to decreased workplace performance, productivity, and increased risk of unemployment[6, 7]. Depression and anxiety are the two most common mental disorders globally, and are therefore also most likely to impact work performance and productivity[8].

Increasing access to mental health support is a key factor for treating mental disorders. Research highlights several important barriers which complicate help-seeking, with mental health related stigma being the most prominent[9]. Stigma can be defined as the convergence of several interrelated components, such as labeling, stereotyping, separation, status loss, and discrimination which occur together[10]. This includes perceived stigma (also known as social stigma) relating to an individual's perception of what others think and feel, and personal stigma (also known as self-stigma) reflecting individual thoughts and attitudes restricting openness about mental health difficulties, increasing risk of social exclusion, and limiting help seeking behavior[11, 12]. In a nationwide US study, over 90% of first responders found stigma as a main barrier to seeking help for themselves[9]. International evidence indicates that experiences of stigma and discrimination lead to decreased use of mental-health related interventions, including workplace-based mental health promotion programmes[13, 14, 15]. Mental health-related stigma can also lead to the breakdown of social connections including avoidance, rejection, and a perception of reduced competence[16]. As a consequence, the person involved may experience lack of career development, reduction of responsibilities, inequity in workplace policies, and exclusion from work integration and social activities. Stigma has also been found to increase the risk of unemployment, job uncertainty, and reduced the likelihood of being hired [17].

Addressing mental health related stigma is a central component of LaMontagne's [18] model for workplace mental health, which integrates preventing harm and reducing risk factors, promoting the positive aspects of work, and management of mental illness. Investing in mental health in the workplace via mental health promotion actions can not only improve mental health on an individual level, but also increase economic productivity[19, 20, 21]. Several workplace-based mental health promotion programmes have been implemented in the EU, with the majority of these being conducted in large companies. This means that interventions are only reaching a small proportion of all employees as the majority (99%) of EU-based workplaces represent Small or Medium size Enterprises (SMEs)[22]. Despite proportionally more people being employed by SMEs in comparison to larger companies, SMEs often lack the financial and/or human resources (HR) support for mental health promotion. Although face-to-face interventions seem to be more effective, research shows that online interventions can be time- and cost-effective, and also easily implementable which can be favourable for small enterprises with presumably limited budgets to implement mental health promotion activities[23].

Although research has shown that stigma can lead to a number of negative consequences and is a barrier for workplace mental health promotion, more insight is required into how best to reduce stigma. A number of intervention studies investigating the effects of anti-stigma initiatives have been conducted during the last 10-20 years, and so far only one systematic review has been published[24]. This review identified 16 intervention studies targeting stigma of mental illness at the workplace. The review included research published between 2004 and 2014 and found support for anti-stigma interventions leading to improved employee knowledge

For peer review only

and supportive behaviour towards people with mental-health problems. They concluded that while the majority of interventions demonstrated a positive effect on employee's attitudes, there remained significant need for improved methodological quality in future evaluations. Specifically, selection bias might have contributed to the positive effects. In particular, one of the main findings indicated that the majority of the interventions were conducted with more highly educated supervisors or in job groups, with more highly educated employees and in the public sector. This reduces the generalizability to most workplaces in other diverse sectors with less educated workers. Consistent with workplace mental health research in general, most of these studies were also conducted in larger organizations, and therefore not providing any knowledge about interventions designed to reduce stigma in SMEs. The currently ongoing intervention project Mental Health Promotion and Intervention in Occupational Settings (MENTUPP Project) aims to contribute to knowledge in this area. A comprehensive online intervention has been developed and is currently being tested in a number of SMEs across European countries and Australia[25]. This review has been conducted as part of the MENTUPP Project to enhance its evidence base.

Therefore, the main aim of this paper was to systematically review the current evidence for interventions focusing on reducing stigma related to mental health problems in SMEs in various sectors. A secondary aim of the review was to investigate the mode of delivery and intensity/duration of interventions.

Methods

Review procedure

A systematic literature search was conducted with a focus on interventions targeting mental health related stigma in the workplace. The review was conducted in accordance with the PRISMA guideline process[26]. Peer-reviewed articles about workplace-based anti-stigma interventions were searched from January 2010 until 14th July 2021 via PubMed, Ovid Medline, PsycINFO, Scopus and Cochrane databases. An additional Google Scholar search was conducted. All results from the database search were uploaded to Covidence (www.covidence.org), an online tool for managing and streamlining systematic reviews.

Study selection

The systematic review was conducted addressing the following inclusion criteria: 1) the sample included employees and/or owners/managers; 2) the intervention at the workplace was aimed to reduce stigma; 3) the outcomes were measured in terms of stigmatization against depression, anxiety and/or other mental health problems; 4) studies had an experimental or quasi-experimental design (including quantitative data); 5) the studies were published in English; 6) the intervention was delivered through the workplace; and 7) the studies were published between January 2010 and July 2021.
Studies were excluded based on the following criteria: 1) no evaluation of the intervention; 2) only qualitative evaluation (e.g. interview or focus group); or 3) no direct measure on stigma (studies with indirect measures of stigma, such as knowledge of mental health, or attitudes towards mentally ill patients, were excluded).

For peer review only

After duplicates were removed, the records were screened by two independent reviewers (GyP, SI) following a two-stage procedure: 1. Inspecting titles and abstracts of the studies, and 2. A full-text read of the articles to assess whether they met inclusion criteria. In the case of disagreement, a consensus was made together with a third researcher (MDT; first author of the study).

Search strategy

The search string was developed by GYP and MDT, reviewed by IS and CL, and subsequently reviewed by a subject librarian at Semmelweis University Hungary (See search keywords in Appendix 1). Terms related to the following themes were used: mental health related terms AND workplace related terms AND stigma-related terms AND intervention related terms.

Included studies

Supplementary figure 1. displays the PRISMA flow diagram which shows the decision points during the screening process.

(SUPPLEMENTARY FIGURE 1)

The PubMed, Ovid Medline, PsycINFO, Scopus and Cochrane databases and Google Scholar were searched resulting in initial identification of 2931 articles. After removal of duplicates (n=215) title screening and abstract review was conducted for 2716 articles, of which 135 were retained for full-text screening, and 19 met criteria for inclusion.

Data extraction

Data extraction by two co-authors for the articles after full-text review included the following and was independently cross-checked by a third reviewer (MDT): (1) Author and year; (2) study design; (3) number of participants at baseline and follow up; (4) gender of participants (5) Target group (6) Sector and Size of organization (7) intervention; (8) intervention intensity; (9) country (10) (Supplementary Table, 1) outcome measure on stigma; (11) evaluation timepoints; (12) main findings (Supplementary Table 2).

The review was conducted according to PRISMA guidelines[26] and registered with PROSPERO: ID: CRD42020191307.

Quality assessment

The methodological quality of each included article was assessed using the Quality Assessment Tool for Quantitative Studies (QATQS) scale[27], based on the following aspects rated from weak to strong: selection bias, design, confounders, blinding, data collection method and drop-out. The global rating was high, in case of 'no weak rating', moderate in case of one 'weak rating', and weak in case of 'two or more weak ratings'. Quality assessment was finalized after two independent reviews by the first and second author of this review, followed by a consensus meeting together with a third independent reviewer GYP.

For peer review only

Patient and Public Involvement

No patient involved.

Results

Study Characteristics

Of the 19 included intervention studies, six were conducted in Canada, five in Australia, four in Great Britain and one each in Germany, Sweden, Spain and Japan. Seven studies used a Randomized Controlled Trial (RCT) study design and the remaining 12 used a quasi-experimental design. An overview of the studies included is presented in supplementary table 1 and 2.

(SUPPLEMENTARY TABLE 1)

(SUPPLEMENTARY TABLE 2)

Sector and size of organization

A total of 19 interventions were used by the included studies, most of which (10/19) were conducted in public sector organizations, or in a mixture of public and private sector workplaces (4/19). Only three studies focused solely on private sector companies, and no sector-specific information was provided in two of the studies. The interventions enrolled different professional groups in varying positions including healthcare workers (2 studies), first responders (3), public servants (2), maintenance staff (2), governmental employees (2), housing association (1), managers, leaders (8).

Five studies provided information on the size of the organizations, the four studies in the private sector enrolled large enterprises with more than 250 employees. Two interventions enrolled a mixture of small, medium and large organizations. No intervention study specifically focused on SMEs.

Quality assessment of the studies

The assessed methodological quality of the included studies varied from weak to strong, with two considered to be of high quality. Two thirds of papers (12/19) were assessed as having moderate quality, most lacking a control group design. Five articles were appraised as weak, a rating driven primarily from low agreement rate and/or high dropout rate (supplementary table 3).

(SUPPLEMENTARY TABLE 3)

The detailed evaluation criteria of the QATQS scale are presented in supplementary table 4.

(SUPPLEMENTARY TABLE 4)

Interventions

Overall, 49% of the interventions used previously developed standardized interventions, including the Mental Health First Aid program, Psychological First Aid, Applied Suicide

For peer review only

Intervention Skills Training, Beyond Blue or Mental Health-Guru, with other interventions being designed or modified to fit a workplace-based context. In terms of implementation, four interventions included in the studies were delivered online, 13 delivered in person, and two were blended interventions (delivered both online and face to face). All programmes used multimodal approaches, which included multiple intervention techniques such as psychoeducation, interactive skills training exercises, and case vignettes/videos of experts with lived experience. Some of the interventions contained specific leadership-focused elements. The most frequent topics were: education about the features and symptoms of mental disorders (special focus on depression and anxiety), warning signs of mental disorders, crisis and suicidal risk and its management, importance of mental health issues in the workplace, and communication strategies for supporting employees with mental health problems.

As a general result we found a significant reduction in stigmatizing attitudes in almost all studies (17/19), using nine different instruments/scales. A detailed overview of study characteristics is presented in Supplementary Table 1 and the main findings of each study are presented in Supplementary Table 2.

Mode of delivery

In the next section we will shortly describe some main features of the 19 studies. First, we present the online interventions, then the face-to-face interventions and finally the blended interventions. Within each category we begin with presenting studies with a RCT design followed by studies with a quasi-experimental or other study designs.

Online interventions

Four out of the 19 studies delivered the intervention in an online format[28 - 31]. Out of the four studies, three found significant positive effects on stigmatizing attitudes, while one intervention did not find a positive effect after the intervention[31]. The average length of these online interventions was 146 minutes, the shortest being 30-45 minutes and the longest six hours. The positive effects were maintained at three[29] and six months follow up[28, 30].

RCT design studies

Griffiths et al investigated the effectiveness of a 1-hour long online mental health programme for employees of governmental organizations (N=507) [28]. Significant reduction measured by the personal subscales of The Depression and Generalized Anxiety Stigma Scales[32, 33] was found post intervention and 6 months follow-up. **Shann et al** delivered an online leadership intervention (N=311) [30]. Even a short, 30-45 mins duration intervention resulted in a significant reduction in stigma scores even at 6-month follow up, which was measured by a 12-item Managerial Stigma Toward Employee Depression Scale[34].

Studies with non RCT design

Paterson et al delivered a 6-hour long online workplace intervention (N=134) [31]. No significant difference in pre and post measures stigma scores between intervention and control group was found, and the methodological quality was rated as weak. The adopted version of King's stigma Scale was used[35]. **Hanisch** et al. delivered a 2-hour digital training for managers (N=48)[29]. The intervention resulted in significant reduction regarding stigmatization toward people with mental health problems, but no control group was enrolled.

For peer review only

The Opening Minds Scale for Workplace Attitudes was used post-training and at 3 months follow-up[35].

Face-to-face interventions

Most of the studies used a face-to-face approach (13 out of 19). The average length of these interventions was 8.6 hours (=516 minutes), the shortest being two hours and the longest 16 hours interventions. Only one intervention did not find a significant positive effect on stigmatizing attitudes[36], and one revealed rebound effect by 3 months follow up[37]. Two further studies did not have a follow-up measurement [38, 39]. The length of the follow up varied between 1 month to 2 years.

Studies with RCT design

Five studies used RCT designs, one rated as a methodologically strong study: **Svensson and Hansson**[40] conducted a 12-hour long training for public sector employees (N=199). A vignette version of the Depression Personal and Perceived Stigma scale[32] showed significant reduction in personal stigma towards people with depression after 6 months and even at 2 years follow up, but no significant changes were found in the control group. Similarly, the other three studies[41, 42, 37] found significant reduction in stigmatizing attitudes in their intervention group post training, and 1 or 2 months follow-up, but no significant changes were found in the control groups. The effects of 3 to 4 hours face to face trainings were measured by the modified version of the Depression Stigma Personal subscale[32], the Opening Minds Scale for Workplace Attitude[35] and the Opening Minds Stigma Scale for Health Care Providers[43]. Fire service line managers (N=106) were randomly assigned to either a 2-day or 12 hour long training group or a control group (1 hour leaflet session)[39]. The locally developed Knowledge and Efficacy about Mental Health Problems scale revealed statistically significant improvements in stigma on mental health pre- and post-training in both training settings, but not in the control group.

Studies with non-RCT design

Four out of five *weak methodological quality* interventions were performed a face-to face non-RCT design.

Bond et al. [44] delivered a 4-hour course for employees in support services (N=284). Significant reduction was found on stigmatizing items measured by an adapted version of Depression Stigma Scale[12] after the course and 6-month follow up. **Kubo** et al[45] delivered a 2-hour long education program (N=91). Right after the intervention, the Japanese version of the Links Perceived Devaluation-Discrimination Scale[10] showed a significant decrease in negative attitudes towards mental health problems, but this difference was not maintained after 1 month. Although there was a long-term (2 years) effect in perceived mental health stigma in **Kristman's et al**[46] 2 year-long quasi-experimental study (N=89), the methodological quality of the study was assessed as weak. **Quinn et al**[47]conducted a 6-hour long training course for telecommunication workers (N=101). Relevant questions gathered from the Scottish Public Attitudes Survey[48] revealed a significant decrease in stigmatizing attitudes between pre and post intervention, however the methodology was rated as weak.

Five other face-to-face studies were rated as having *moderate methodological quality*: **Dobson** et al[49] (N=1292) and **Szető et al** (N=5598) investigated the effects of a 4- and 8-hour long

For peer review only - http://bmjopen.bmj.com/site/about/guidelines.xhtml

stigma reduction program for frontline workers and managers[50] . In both studies, the Opening Minds Scale for Workplace Attitudes[35] showed a significant reduction in stigma for the total scale and all the subscales between pre- and post- intervention and 3-month follow up in both groups. In their longitudinal cohort study, **Hamann** et al [38] delivered a 1-1.5-day long face-to-face educational workshop for leaders and HR department employees (N=580). Post-intervention, the Depression Stigma Personal Subscale[32] showed a significant decrease, but no follow-up measure was performed. On the other hand, reduction in stigma was not significant in a 1-hour training followed by an 4-hour gate-keeper training for Australian Mates in Mining co-workers (N=1275) and 117 supervisors[36]. Mental health stigma was measured by Perceived Stigma Scale[51].

Blended studies

Both of the blended design studies used randomized designs. In **a** strongly rated methodology study by Moll et al, mental health literacy training was delivered to healthcare workers (N=192) in either face-to face or blended setting [52]. Both interventions resulted in a significant reduction of stigmatized beliefs, but a longer effect was seen by the blended intervention at 6-month follow up, which was measured by the Opening Minds Scale for Health Care Providers[43]. In a study by Reavley at al of 608 public sector employees were randomized into different interventions: two MHFA and PFA online courses and a blended MHFA one[53]. Significant reduction in stigma scores were found in each intervention groups and the Personal Stigma Scale[12] showed no significant difference between online and blended courses.

Discussion

The main aim of this systematic review was to identify and evaluate the effectiveness of different workplace-based anti-stigma interventions, focusing on reducing stigmatizing attitudes and discrimination of people with mental illness. The review included interventions that were delivered to employees and employers. A specific focus was placed on Small and Medium Enterprises.
Nineteen articles met the inclusion criteria and we found an overall positive effect for most of the interventions irrespective of the modeof delivery. Three of the four studies using online interventions found positive effects. Among the 13 face-to-face interventions, only one study did not find an effect, although a few studies only found short term effects. This finding appears to indicate that online anti-stigma interventions can be just as effective as face-to-face interventions. Similarly, a study comparing training for managers to improve their confidence in supporting the mental health of their employees found that both the online and face-to-face version to be effective[23]. As for the intensity of the intervention, we can conclude that the average length of online interventions was substantially shorter compared to those delivered face-to-face (146 min vs 516 min on average).

The finding that online interventions might be just as effective as face-to-face interventions was also confirmed by two further randomized control studies identified in this review. Reavley et al[53] found no significant difference between the effectiveness of blended and purely online interventions on stigmatizing attitudes, and a longer lasting positive effect was found in a blended intervention compared to its face-to-face version in another study[52]. These results underline the possible benefits of online interventions over the conventional face-to-face approaches: online interventions are shorter, need no presence of the professionals/trainers, and they have particular potential for the workplace as they can be tailored to participant or

For peer review only

workplace needs (i.e., can be used anytime during the day), which may also have favourable cost implications. These features make them especially attractive for SMEs as they typically have fewer resources for implementing workplace mental health interventions. Online interventions can also be beneficial during public health emergencies (such as the Covid-19 pandemic) when face-to-face contact is reduced or not possible.

We can conclude that the quality of the interventions has improved since Hanisch et al.'s review[24], having only three overlapping studies with this previous review (39, 40, 47). We identified studies with larger sample size and longer lasting effects. Our review also confirms the findings of the previous review with more studies with higher methodological quality. However, in this review the majority of the identified studies did not have a control group and the drop-out rate in some studies was high. Only two of the nineteen studies were rated to have strong methodological quality. The majority of the programmes used a multitude of intervention techniques targeting both employees and leaders, which may have made the intervention more effective, but produces difficulties in terms of identifying the most effective elements for stigma-reduction.

With regard to evaluation aspects, 14 studies included follow-up measurements after the intervention, with the duration varying from 1 month to 2 years. Most of the studies used a 1 to 6-month follow up, only two programs followed their participants for 2 years, and both found that the effects were maintained. A few studies however, reported only short-term effects. It remains unclear why some interventions demonstrate long-term effects while other studies only achieved short term effects. More studies with longer follow-up time and more studies with more details about the content of the intervention are needed to investigate this further.

Despite the overall positive outcomes on stigmatizing attitudes by the reviewed studies, it would be important to know if employees actually experience a reduction in exposure to mental health related stigma from their colleagues and managers following the interventions. Measurement tools assess changes in attitudes that do not always translate into differences in behavior and other measures should also be applied in these studies, such as the willingness to seek or offer help.
One of the 2 studies which did not find a significant reduction in stigmatizing attitudes after the intervention, investigated the effects of a 6-hour long online training programme[31]. Authors concluded that the stigma questionnaire[33] used in their evaluation may not have been sensitive enough to capture improvement in mental health related stigma in the workplace context. Similarly, a non-validated stigma-measuring scale could be the reason of another intervention which seems to have no significant reduction in stigma scores[36].

Although our primary aim was to review changes in mental health related stigma, other results are also noteworthy. For example, interventions were also found to contribute to increased mental health literacy[52, 41] and intention to seek help[28]. Increased resilience [49, 50] and help-seeking behavior[28, 52] were also observed, confirming previous findings by Hanisch et al[23].

Workplace-based mental health stigma-reduction programs appear to have very similar key objectives and approaches, although we noted a tendency to use different evaluation approaches using different scales. The use of appropriate, psychometrically sound scales to assess stigma is crucial and facilitates comparison of findings. Both of the interventions [31, 36] with no significant reductions in stigma scores applied scales that may not have been sensitive enough in workplace settings. Moreover, some researchers used semi-structured interviews or primarily

For peer review only

qualitative methods for evaluating program effectiveness meaning they were excluded from our review, although these also found a reduction in participants' stigmatizing attitudes[54].

In sum, our main objective was to review effective workplace-based interventions for addressing mental health related stigma with a particular focus on SMEs. Unfortunately, our results did not entirely meet our expectations, as none of the reviewed interventions targeted SMEs specifically. Possible reasons behind this may be due to data protection reasons as limited data on the exact size and type of the organisations were noted. Most of the interventions were conducted in larger companies or public organizations, and therefore it is difficult to determine their feasibility in smaller enterprises with smaller numbers of employees and supervisors. However, we identified positive effects in studies where differently sized companies participated. Stigma-reduction in SME workplaces therefore remains unaddressed, although our review did add some new perspectives for smaller enterprises.

Our purpose to review interventions with appropriate methodology has produced rather positive results. The reviewed papers indicate that the included interventions produced significant reductions in stigmatizing attitudes for both employees and managers, and despite variation in methodology, common conclusions could be drawn.

Limitations

Notwithstanding the positive results of this review, several limitations should be mentioned. Only English language articles were included from five electronic databases, but we did not use occupational health databases for primary literature.

We have identified a clear dominance of interventions targeting higher educated white collar employers and employees, inhibiting the generalizability of effectiveness to less educated or blue-collar employees. In addition, all studies were conducted in either European countries, North-America, Australia or Japan therefore not representing experiences from other parts of the world, with lower economic status. Only studies with quantitative measurement were included in this review, however studies with interview or focus group designs could provide important additional information. Similarly, we did exclude studies with no direct measure on stigma, however attitudes towards mentally ill patients and knowledge of mental health are important factors of stigmatizing behavior. Given the diverse study designs and outcome measures, no meta-analysis was possible to conduct.

Conclusions

A large proportion of the workforce could benefit from workplace-based interventions aimed at reducing mental health related stigma. Although we did not find interventions focusing specifically on SMEs, we can derive important findings from our review. Online anti-stigma interventions could have several benefits for smaller enterprises, they are shorter, and appear to have the same positive effects on stigmatizing attitudes as face-to-face interventions. These could be very important factors for professionals when trying to choose an intervention for their company.

Furthermore, investigations of the feasibility of these programs in smaller enterprises with less resources are needed, and more studies should go beyond measuring only attitudes.

Contributorship Statement

For peer review only

MDT, GYP, SI and EA conceived the idea for the study. MDT, GYP, SI, BA and CL planned the study design. MDT, SI and GYP carried out the search and quality assessments with input from BA and CL. MDT and GYP wrote the first draft of the report with input from BA, JCS, HR, GC, SS, NF, GQ, FT, VR, SM and ACP. All authors contributed to the interpretation of findings and critical revision of the manuscript. All authors approved the final version of the manuscript for submission, MDT submitted the manuscript.

Competing interests

The authors declare no competing interests.

Funding

This work was supported by the European Union's Horizon 2020 Research and Innovation Programme grant number 848137.

Data sharing statement

No additional data available.

Ethics approval

Ethical approval was given by the Hungarian Medical Research Council, reference number: IV/10156-3/2020/EKU.

References

[1] Flores EC, Fuhr DC, Bayer AM et al. Mental health impact of social capital interventions: a systematic review. *Soc Psychiatry Psychiatr Epidemiol* 2018;53(2): 107-119.

[2] Leka S, Jain A, Iavicoli S et al. An evaluation of the policy context on psychosocial risks and mental health in the workplace in the European Union: achievements, challenges, and the future. *BioMed research international*, *2015*.

[3] Johnston DA, Harvey SB, Glozier N et al. The relationship between depression symptoms, absenteeism and presenteeism. *J Affect Disord* 2019;256: 536-540.

[4] Bryan ML, Bryce AM, Roberts J. Presenteeism in the UK: Effects of physical and mental health on worker productivity. The Sheffield Economic Research Paper Series (SERPS) 2020; 2020005(2020005).

[5] Niedhammer I, Bertrais S, Witt K. Psychosocial work exposures and health outcomes: a meta-review of literature reviews with meta-analysis. S*cand J Work Environ Health* 2021;47(7):489–508.

[6] Bubonya M, Cobb-Clark DA, Wooden M. Mental health and productivity at work: Does what you do matter? *Labour Econ* 2017;46: 150-165.

[7] Brouwers EP. Social stigma is an underestimated contributing factor to unemployment in people with mental illness or mental health issues: position paper and future directions. *BMC Psychol* 2020;8(1):1-7.

[8] WHO, 2017: Depression and Other Common Mental Disorders: Global Health Estimates. Geneva: World Health Organization; 2017. Licence: CC BY-NC-SA 3.0 IGO.

[9] Drew JM, Martin S. A National Study of Police Mental Health in the USA: Stigma, Mental Health and Help-Seeking Behaviors. *J Police Crim Psych* 2021;36: 295–306.

[10] Link BG, Phelan JC. Conceptualizing stigma. *Annu Rev Sociol* 2001;27(1): 363-385.

[11] DeFreitas SC, Crone T, DeLeon M et al. Perceived and personal mental health stigma in Latino and African American college students. *Front Public Health* 2018;6: 49.

[12] Griffiths KM, Christensen H, Jorm AF et al. Effect of web-based depression literacy and cognitive–behavioural therapy interventions on stigmatising attitudes to depression: Randomised controlled trial. *Br J Psychiatry* 2004;185(4): 342-349.

[13] Arnaez JM, Krendl AC, McCormick BP et al. The association of depression stigma with barriers to seeking mental health care: A cross-sectional analysis. *J Ment Health* 2020;29(2):182-190.

[14] Fox AB, Smith BN, Vogt D. How and when does mental illness stigma impact treatment seeking? Longitudinal examination of relationships between anticipated and internalized stigma, symptom severity, and mental health service use. *Psychiatry Res* 2018;*268*: 15-20.

[15] Haugen PT, McCrillis AM, Smid GE et al. Mental health stigma and barriers to mental health care for first responders: A systematic review and meta-analysis. *J Psychiatr Res* 2017;94: 218-229.

[16] Couture SM, Penn D. Interpersonal contact and the stigma of mental illness: A review of the literature. *J Ment Health* 2003;12(3): 291–305.

[17] Krupa T, Kirsh B, Cockburn L, Gewurtz R. Understanding the stigma of mental illness in employment. *Work* 2009;33(4): 413-425.

[18] LaMontagne AD, Martin A, Page KM et al. Workplace mental health: Developing an integrated intervention approach. *BMC Psychiatry* 2014;14(1): 131.

[19] Pinheiro M, Ivandic I, Razzouk D. The economic impact of mental disorders and mental health problems in the workplace. In Mental Health Economics (pp. 415-430). Springer, Cham, 2017.

[20] McDaid D, King D, Parsonage M. In: Knapp M, McDaid D, Parsonage M (eds). Mental health promotion and prevention: the economic case. London: Department of Health, 2011:20-1.

[21] Thorpe K, Chénier L. Building mentally healthy workplaces: perspectives of Canadian workers and front-line managers. Ottawa: Conference Board of Canada, 2011

For peer review only

[22] European Commission (2022): https://ec.europa.eu/growth/smes/sme-definition_en. Downloaded: 15th February, 2022.

[23] Gayed A, Tan L, LaMontagne AD et al. A comparison of face-to-face and online training in improving managers' confidence to support the mental health of workers. *Internet Interv* 2019;18: 100258.

[24] Hanisch SE, Twomey CD, Szeto AC et al. The effectiveness of interventions targeting the stigma of mental illness at the workplace: a systematic review. *BMC Psychiatry 2016*;16(1): 1-11.

[25] Arensman E, O'Connor C, Leduc C et al. Mental Health Promotion and Intervention in Occupational Settings: Protocol for a Pilot Study of the MENTUPP Intervention. *Int J Environ Res Public Health* 2022;19(2): 947.

[26] Page MJ, Moher D, Bossuyt PM et al. PRISMA 2020 explanation and elaboration: Updated guidance and exemplars for reporting systematic reviews. *BMJ*, 2021; 372: n160.

[27] Ciliska D, Miccouci S, Dobbins M. Effective public health practice project. quality assessment tool for quantitative studies. Hamilton, On: Effective Public Health Practice Project 1998.

[28] Griffiths KM, Bennett K, Walker J et al. Effectiveness of MH-Guru, a brief online mental health program for the workplace: A randomised controlled trial. *Internet inter* 2016;6: 29-39.

[29] Hanisch SE, Birner UW, Oberhauser C et al. Development and evaluation of digital game-based training for managers to promote employee mental health and reduce mental illness stigma at work: Quasi-experimental study of program effectiveness. *JMIR Ment Health* 2017;4(3): e31.

[30] Shann C, Martin A, Chester A et al. Effectiveness and application of an online leadership intervention to promote mental health and reduce depression-related stigma in organizations. *J Occup Health Psychol* 2019;24(1): 20.

[31] Paterson H, Todorova GK, Noble K et al. Evaluation of Headtorch WORKS as a workplace intervention for improved support and understanding of co-workers with poor mental health and well-being. *Eur J Work Organ Psychol* 2021; 1-12.

[32] Griffiths KM, Christensen H, Jorm AF. Predictors of depression stigma. *BMC Psychiatry* 2008;*8*(1): 1-12.

[33] Griffiths KM, Batterham PJ, Barney, L et al. The Generalised Anxiety Stigma Scale (GASS): psychometric properties in a community sample. *BMC Psychiatry*, 2011;11(1): 1-9.

[34] Martin A. Individual and contextual correlates of managers' attitudes toward depressed employees. *Human Resource Management* 2010;49(4): 647-668.

[35] King M, Dinos S, Shaw J et al. The stigma scale: Development of a standardised measure of the stigma of mental illness. *Br J Psychiatry* 2007;190: 248–254.

For peer review only - http://bmjopen.bmj.com/site/about/guidelines.xhtml

[35] Szeto AC, Luong D, Dobson KS. Does labeling matter? An examination of attitudes and perceptions of labels for mental disorders. *Soc Psychiatry Psychiatr Epidemiol* 2013;48(4): 659-671.

[36] Tynan RJ, James C, Considine R et al. Feasibility and acceptability of strategies to address mental health and mental ill-health in the Australian coal mining industry. *Int J Ment Health Syst* 2018;12(1): 66.

[37] Eiroa-Orosa FJ, Lomascolo M, Tosas-Fernández A. Efficacy of an Intervention to Reduce Stigma Beliefs and Attitudes among Primary Care and Mental Health Professionals: Two Cluster Randomised-Controlled Trials. *Int J Environ Res Public Health* 2021;18(3): 1214.

[38] Hamann J, Mendel R, Reichhart et al. A "Mental-Health-at-the-Workplace" Educational Workshop Reduces Managers' Stigma Toward Depression. *J Nerv Ment Dis* 2016;204(1): 61-63.

[39] Moffitt J, Bostock J, Cave A. Promoting well-being and reducing stigma about mental health in the fire service. *J Public Ment Health* 2014;12(2): 103-113.

[40] Svensson B, Hansson L. Effectiveness of mental health first aid training in Sweden. A randomized controlled trial with a six-month and two-year follow-up. *PloS one* 2014;9(6): e100911.

[41] Dimoff JK. Mental health awareness training (MHAT): the development and evaluation of an intervention for leaders. *Int J Stress Manag* 2016;23(2): 167–189.

[42] Dobson KS, Markova V, Wen A et al. Effects of the Anti-stigma Workplace Intervention "Working Mind" in a Canadian Health-Care Setting: A Cluster-Randomized Trial of Immediate Versus Delayed Implementation. *Can J Psychiatry* 2021;66(5): 495-502.

[43] Kassam A, Papish A, Modgill G et al. The development and psychometric properties of a new scale to measure mental illness related stigma by health care providers: the Opening Minds Scale for Health Care Providers (OMS-HC). *BMC Psychiatry* 2012;12(1): 1-12.

[44] Bond KS, Cottrill, FA, Mackinnon, A et al. Effects of the Mental Health First Aid for the suicidal person course on beliefs about suicide, stigmatising attitudes, confidence to help, and intended and actual helping actions: an evaluation. *Int J Ment Health Syst* 2021;15(1):1-12.

[45] Kubo H, Urata H, Katsuki R et al. Development of MHFA-based 2-h educational program for early intervention in depression among office workers: A single-arm pilot trial. *PloS One* 2018;13(12): e0208114.

[46] Kristman VL, Lowey J, Fraser L et al. A multi-faceted community intervention is associated with knowledge and standards of workplace mental health: the Superior Mental Wellness@ Work study. *BMC Public Health* 2019;19(1): 638.

[47] Quinn N, Smith M, Fleming S et al. Self and others: the differential impact of an anti-stigma programme. *Stigma Res Action* 2011;1(1): 36-43.

[48] Braunholtz S, Davidson S, King S. Well? What do you think? The second national Scottish survey of public attitudes to mental health, mental well-being and mental health problems. Edinburgh: Scottish Executive, 2004.

[49] Dobson KS, Szeto A, Knaak S. The Working Mind: A meta-analysis of a workplace mental health and stigma reduction program. *Can J Psychiatry* 2019;64(1): 39S-47S.

[50] Szeto A, Dobson KS, Knaak S. The road to mental readiness for first responders: a meta-analysis of program outcomes. *Can J Psychiatry* 2019;64(1): 18S-29S.

[51] Kelly BJ, Stain HJ, Coleman C et al. Mental health and well-being within rural communities: the Australian rural mental health study. *Aust J Rural Health* 2010;18:16–24.

[52] Moll SE, Patten S, Stuart H et al. Beyond silence: a randomized, parallel-group trial exploring the impact of workplace mental health literacy training with healthcare employees. *Can J Psychiatry* 2018;63(12): 826-833.

[53] Reavley NJ, Morgan AJ, Fischer JA et al. Effectiveness of eLearning and blended modes of delivery of Mental Health First Aid training in the workplace: randomised controlled trial. *BMC Psychiatry* 2018;18(1): 1-14.

[54] Ross V, Caton N, Gullestrup J et al. Understanding the barriers and pathways to male help-seeking and help-offering: a mixed methods study of the impact of the Mates in Construction Program. *Int J Environ Res Public Health* 2019;16(16): 2979.

For peer review only

Supplementary table 1. Overview of study characteristics

First author/ year	Study Design	Population at baseline and follow up	Gender at baseline	Target group	Sector/ Size of organization	Intervention	Intervention Intensity	Country
**Bond et al, 2021**	longitudinal cohort study	284 pre<br>98 post	212 female<br>72 male	support services, police, educators and general community networks	public sector	Mental Health First Aid for the Suicidal Person course	4-hours course	Australia
**Dimoff 2016**	controlled study active vs wait list	183 pre<br>142 post	77 female<br>65 male	Leaders in telecommunication companies	large company, private sector	Mental health awareness training	3 hours training	Canada
**Dobson et al, 2021**	cluster-randomized trial	123 pre<br>101 post	115 female<br>8 male	office workers<br><br>kitchen and maintenance staff	large company, public sector	The Working Mind program:<br>• trained facilitators,<br>• workshop manuals,<br>• contact-based videos<br>• discussion exercises,<br>• personal goal setting.<br>Participants allocated to immadiate or delayed intervention groups.	4-hours group program	Canada
**Dobson et al.,<br><br>2019**	open trial methodology	1292 pre<br>1155 post	male 419<br>female 719	government, education, health, energy supervisors and frontline staff	public sector	The Working Mind Program<br>• trained facilitators,<br>• workshop manuals,<br>• contact-based videos<br>• discussion exercises,<br>• personal goal setting.<br>"train-the- trainer" model	Two versions:<br>4-hour group program for frontline workers<br><br>8-hour program for managers	Canada
**Eiroa-Orosa et al, 2021**	cluster randomized-	371 pre<br>260 post	314 female<br>57 male	primary health and mental health care professionals	public sector	awareness-raising intervention<br><br>The Targeted, Local, Credible, Continuous Contact (TLC3)	**4 workshops**<br>1. Training: pedagogy and contact (face-to face +video) – 4 hours	Spain

	controlled trial			administrative officers, general practitioners, odontologists, nurses, psychiatrists, psycholhologists, and social workers.		methodology adapted to the Catalan healthcare context	2. self-diagnosis and prioritization – 4 hours<br>3. self-organized activities<br>4. follow-up session	
**Griffith et al 2016**	Randomised controlled trial	507 pre<br>386 post	MH-guru: male: 29%, female: 70%;	multi-departmental government workplace	public sector	online depression and anxiety educational workplace induction program ("Mental Health Guru"): two modules: depression and General anxiety disorder	1 hour long online depression and anxiety educational program (1 module/week, 30min/module)	Australia
**Hamann et al., 2016**	Longitudinal cohort study	580 pre	210 women<br>370 men	Leaders, members of the workers' council, workers in HR department	not specified companies (n=30)	"Mental-health-at-the-workplace" educational workshop	1-1,5 days training	Germany
**Hanisch et al., 2017**	Longitudinal cohort study	48 pre	92% male, 8% female	Leaders	private sector – large enterprise	"Leadership Training in Mental Health Promotion" (LMHP), a digital game-based training program for leaders which is combining games and simulations in a virtual environment.	1.5- 2 hours long single session	UK
**Kristman et al., 2019**	quasi-experimental	89 pre<br>61 post	59 male<br>24 female	Leaders in HR, occupational health and safety management	public and private sector, different size companies	Multi-faceted:<br>1. "Standard to Action" training program designed to help employers implement the Standard in	2 years	Canada

						their workplaces – 6 sessions;<br>2. Education sessions: MH First Aid sessions;<br>3. Social marketing campaign including a photovoice exhibit.		
**Kubo et al, 2018**	Single arm pilot trial	91 pre<br>83 post	male 77%<br>female 23%	office workers	no specific informatio n	“Mental Health First Aid” (MHFA) training program modified for workplace settings.	2-hour training course	Japan
**Moffitt et al, 2014**	random allocation design	106 pre<br>89 post	N/A	fire service line managers	public sector	Participants randomly assigned to:<br>• Looking after Wellbeing at Work” (LWW)<br>• Mental Health First Aid (MHFA)<br>• leaflet session (LS).	LWW- 2days<br>MHFA – 12 hours<br>LS- 1 hour	UK
**Moll et al, 2018**	randomise d, parallel-group trial	192 pre<br>167 post<br>150 by 6 mo follow up	female 88.5%<br>male 11.5%	Healthcare workers	public sector	“Beyond Silence” (Beyond Silence program includes a contact-based educational approach)<br>Mental Health First Aid training	comprising 6 in-person, 2-h sessions + 5 online sessions co-led by employees who personally experienced mental health issues<br>standardised 2-days training program	Canada

**Paterson et al, 2021**		134 pre<br>57 post	not specified	not specified	public and private, different size companies	Headtorch WORKS - mental health and well-being intervention	3 online episodes + discussion group<br>6 hours<br>original filmed drama and specialist documentary	UK
**Quinn et al, 2011**		101 pre<br>87 post	77% male<br>23% female	housing association and telecommunication workers	public and private sector	Training course on mental health awareness	One day training (6 hours each)<br><br>combination of service user narratives, experiential group learning, and didactic teaching approaches.	Scotland
**Reavley et al., 2018**	Randomized controlled trial	608 pre<br>289 post	449 female<br>159 male	public servants	public sector	Participants randomized to<br><br>-eLearning MHFA,<br><br>-blended MHFA<br><br>-PFA eLearning	- 6-hour eLearning MHFA online course<br>- 6-hour eLearning MHFA plus 4-hour face-to-face session<br>- 4-hour eLearning PFA online course	Australia
**Shann et al., 2018**	Randomized controlled trial	311 pre<br>196 post	148 male<br>163 female	Leaders	public<br>private<br>non- profit<br>other sectors 1%	"Beyondblue" online materials for leaders:<br>Main focus on depression:<br>-written information,<br>-video clips of organizational leaders speaking<br>about mental health in the workplace,<br>-interactive exercises in which participants can calculate the cost of untreated depression and the	30 – 45 min	Australia

						specific risk factors in their organization.		
**Svensson and Hansson, 2014**	Randomized controlled trial	416 pre<br>277 post	151 female<br>48 male	Not specified employees	public sector	Mental Health First Aid training	12 hours course, spread over two days	Sweden
**Szeto et al. 2019**	non-randomized quasi-experimental	5598 pre<br>4649 post<br><br>Frontline staff 75.8% (3,449)<br>Supervisory staff 26.4% (1,210)	male 55.9%<br>female 44.1%	Corrections 9.0% (418)<br>Emergency Services (9-1-1) 3.9% (192)<br>Fire Services 17.7% (821)<br>Police Services 56.5% (2,623)<br>Paramedics 13.0% (605)	public sector	"Road to Mental Readiness for First Responders" program (R2MR)<br>**3 main components**:<br>stigma reduction through video contact-based education,<br>the Mental Health Continuum Model, and<br>"Big 4" coping and resilience skills.<br>Additional skills for supervisors.	4-hour program for employees<br><br>8-hour program for supervisors	Canada
**Tynan et al. 2018**	Non-Randomised controlled trial	1275 pre<br>1163 post<br><br>Supervisor:<br>117 pre<br>114 post	1014 male;<br>135 female;<br><br>Supervisor training:<br>92 male;<br>10 female;<br>12 not specifed.	Manager<br>Professional<br>Trades worker<br>Machinery operator Admin or other	private sector, medium and large	"Working Well Mental health Program":<br><br>peer-based, multi-component mental health and suicide prevention program<br><br>supervisor training	- 1 hour 'general awareness training' (GAT),<br>- 4 hours of 'gate-keeper training',<br>-2-day 'Applied Suicide Intervention Skills Training' (ASIST) for key workers.	Australia

Supplementary table 2. Study findings

**Results from Randomized Controlled Trials**				
**First author/year**	**Outcome measure on stigma**	**Evaluation timepoints**	**Main findings**	
Dimoff 2016	Depression Stigma personal Scale (DSS)	pre training<br>post training<br><br>2 months follow up	Significant improvements in stigmatizing attitudes were also observed for the intervention group from T1 (M = 2.98, SD = 0.39) to T2 (M =3.25, SD = 0.37; t (87) = -5.60, p < .001) and from T1 to T3 (M = 3.20, SD = 0.42; t (87) = -4.06, p < .001).<br>No significant improvements in attitude were observed for the intervention group between T2 and T3.	
Dobson et al, 2021	Opening Minds Scale for Workplace Attitudes (OMS-WA)	pre, post training<br>3 months follow up	Stigma scores on the OMS-WA revealed a significant time effect, F(2/154) = 16.33, P < 0.001. There was also a significant group effect, F(1/76) = 16.23, P <0.001, but the interaction effect was not statistically significant, F(2/154) = 1.02, P = 0.362.<br>Pairwise comparison analyses revealed a significant pre- to postreduction in stigma for both the immediate, t(154) = 3.22, P = 0.004, and the delayed group, t(154) = 4.12, P < 0.001.<br>Significant reduction in stigma from pre- to posttest, which was maintained to the time of the follow-up assessment.	
Eiora-Orosa et al, 2021	Opening Minds Scale for Health Care Providers (OMS-HC)<br><br>Beliefs and Attitudes towards Mental Health Service users' rights	pre,<br>1 month follow-up<br>3 months follow up	At baseline statistically significant difference between the intervention and control groups in the total score of the OMS-HC scale (t = 2.138, p < 0.05)<br><br>Statistically significant decreases were seen between baseline and first follow-up for the OMS-HC total score (t = 2.813, p < 0.01)<br><br>The general linear models showed a statistically significant drop between the first observation and the second for the OMS-HC disclosure scores with statistically significant effects (F = 26.881, p < 0.001)<br><br>Reductions in both PC and MH professionals' stigmatising beliefs and attitudes were found in the 1-month follow-up, although a 'rebound effect' at the 3-month follow up was detected.	

For peer review only

Griffith et al. 2016	depression and anxiety personal stigma scale (DSS-personal) (GASS-personal)	baseline,<br>1 week post-intervention<br>6-month follow-up	MH-Guru group showed significantly greater ↓in depression and anxiety personal stigma. Between group effect sizes in stigma for depression were − 0.56 and − 0.47 at post-test and 6-months respectively and − 0.42 at both time points for anxiety (p<.001)<br><br>DSS (Mean, SD)<br>MH-Guru: before: 7.1 (4.9) after: 3.9 (3.8) follow up 4.2 (3.8)<br>Control: Before: 7.3 (5.2) after: 6.8 (5.0) follow up: 6.6 (5.2)<br>F (2, 294.1)=2.5 *P*<.001<br><br>GASS (mean, SD)<br>MH-guru: before: 5.1 (5.1) after: 2.5 (3.9) follow up: 5.1 (0.48)<br>control: before: 4.9 (5.6) after: 5.0 (5.3) follow up: 4.9 (0.34)<br>F (2, 286.1)=19.8 p <.001	
Moffitt, 2014	locally developed "Mental Health Stigma Questionnaire"	pre, post intervention	The LWW and MHFA courses were associated with statistically significant improvements in stigma on mental health.<br>The comparisons showed no significant difference at Time 2 between the LWW and MHFA groups on stigma scale (z= 0.57, p = 0.57, r = 0.07).	
Reavley et al, 2018	Personal Stigma Scale (PSS)	Pre, post training	Those in the blended and eLearning MHFA groups were significantly more likely to show reduced stigma towards people with depression and PTSD than those in the PFA eLearning group.<br>No significant differences between the MHFA eLearning and blended courses.	
Shann et al. 2018.	Managerial Stigma Toward Employee Depression Scale<br>- Affective Stigma Subscale,<br>- Behavioral Stigma Subscale,	pre – post intervention<br>6 month follow up	Significant reductions in behavioral and affective depression-related stigma scores among leaders who completed the intervention, same reduction at 6 months.<br><br>One-way multivariate analysis of covariance showed a statistically significant difference in survey stigma between experimental and control groups, V =.09, F(3, 189)= 6.26, p <.001.<br>Follow-up univariate analyses of variance showed that at posttest, affective stigma was significantly different between groups, F(1, 191) = 14.55, p <.001.	

For peer review only

	- Cognitive Stigma Subscale		The experimental group had lower affective stigma scores (M= 9.42, SEM= .24) at postsurvey compared with the control group (M= 10.51, SEM= .16).	
Svensson and Hansson, 2014	vignette version of the Depression Personal and Perceived Stigma scale (DSS)	pre<br>6 months and<br>2 years follow up	Significant reduction in depression personal stigma after 6 months follow up. Intervention group: pre: 35,8 (5,2) post: 36,3 (4,8). Control group: pre: 36,4 (4.5) post: 35.4 (5.3). F=6,3 p<.05, effect size:0,29.<br><br>The training after two years still have a notable impact on the awareness of mental health and its treatment.	
**Results from quasi-experimental or pre-post design studies**				
Bond et al., 2021	9 statements designed to measure stigmatising attitudes based on Depression Stigma Scale (DSS) – suicide vignette	pre, post training,<br>6 month follow-up	Reductions in scores on „weak not sick" item after the course and at follow-up (t(275.6)=8.89, p<.0001 and t(132.7)=2.66, p<.0001. Changes in means of „Dangerous/unpredictable"item from pre-course were signifcant both postcourse and at follow-up (t(267.0)=11.74, p<.0001 and t(125.5)=3.81, p=0.0002, respectively).	
Dobson et al.,<br><br>2019	- Stigma towards mental health problems (OMS-WA)	pre program<br>post program<br>3-month follow-up period	The results of the mixed-model analysis revealed statistically significant ↓ in stigma for the total scale, coefficient = .167, SE = .08, z = 20.72, P < 0.001, and all subscales (all Ps < 0.001).<br>The mixed-model analysis for the pre- to post- change on the resiliency skills scale revealed statistically significant improvement at the 95% level of confidence (P < 0.001).	
Hamann et al., 2016	Depression Stigma Scale (DSS)	Pre, post training	significant ↓in personal stigma (mean [SD], 15.5 [3.8]; paired t-test: t = 27.6, p < 0.001)	
Hanisch et al, 2017	-Stigma towards mental health problems (OMS-WA)	pre, post-training,<br>3-month follow-up	Positive changes on attitudes toward people with mental health problems (*P*<.01).	
Kristman et al., 2019	-Perceived mental health stigma on the workplace	presurvey<br>postsurvey - 2 yrs	Intervention group - pre: mean (SD)= 1.84 (0.74) post: 1.71 (0.64) MD: -0.13, 0.11)	

	Questions derived from Workplace Mental Health in Canada survey.		Significant difference in perceived mental health stigma btw intervention and non-intervention group:<br><br>Intervention group: mean (SD): 1.52 (0.57) vs. Non-intervention group: 2.00 (0.63), MD: -0.48, 95% CI	
Kubo et al. 2018	-Stigma towards mental health problems:<br>Link's Devaluation-Discrimination Scale	pre-program, post-program,<br>1 month follow up	↓ after the program (before: mean (SD)=28.29 (4.9), after: mean (SD) 26.11 (5.36) p=0.003),<br>no difference 1 month after the program. mean (SD): 27.26 (5.78)	
Moll et al. 2018	Stigma towards mental health problems – health care (OMS-HC)	presurvey<br>Postsurvey<br>3-mo assessment, 6-mo follow-up	Stigmatized beliefs significantly ↓ in both programs.<br><br>In the stigma analysis, no interactions for treatment arm by time were observed at 3 mo (beta = 0.21, z = 0.22, P = 0.83); although, a possible trend for superior outcomes for Beyond Silence was seen at 6 mo (beta = 1.72, z = 1.7, P = 0.089). To explore whether the anti-stigma effects of Beyond Silence might be more persistent than those of MHFA, a model describing changes from 3 to 6 mo was fit, revealing a significant treatment by time interaction (beta = 1.89, z = 2.09, P 1⁄4 0.037).	
Paterson et al, 2021	Adopted version of King's stigma scale[1]	pre, post intervention	There was no significant difference in the pre/post-intervention change in stigma score between the experimental and control groups.	
Quinn et al, 2011	questions gathered from the Scottish Public Attitudes Survey[2]	pre, post intervention	Attendance at the workshop reduced the level of stigmatizing attitudes for both first (t = 11.939, df = 86, p < 0.0005) and third (t = 3.535, df = 86, p = 0.001) person views. The workshop was associated with a more marked reduction in stigmatizing attitudes	

[1] King, M., Dinos, S., Shaw, J., Watson, R., Stevens, S., Passetti, F., . . . Serfaty, M. (2007). The stigma scale: Development of a standardised measure of the stigma of mental illness. British Journal of Psychiatry, 190(MAR.), 248–254

[2] Braunholtz, S., Davidson, S., & King, S. (2004). Well? What do you think? The second national Scottish survey of public attitudes to mental health, mental well-being and mental health problems. Edinburgh: Scottish Executive.

For peer review only

			expressed by first compared with third person views.	
Szeto et al. 2019	Stigma towards mental health problems (OMS-WA)	pre-program, post-program, 3 month follow up	↓in stigma were observed for the total scale and all subscales. before: 1.97 (SD: 0.47). After: 1.85 (SD: 0.49) coeff: 0.123 SE: 0.008 z: 15.87 $p<0.001$ Reductions in stigma were maintained until the final follow-up for the total scale. coeff: - 0.002 SE: 0.012 z: - 0.13 $p=0.899$	
Tynan, 2018	-Mental health stigma, measured by *a perceived stigma* scale[3]	pre-test post-test 10 months follow up	Trend towards a decrease in stigma across both control and intervention sites, however the effect of time or treatment was not signifcant ($p > 0.01$)	

Key. DSS: Depression Stigma Scale. GASS: The Generalised Anxiety Stigma scale, OMS-WA: Opening Minds Scale for Workplace Attitudes, OMS-HC: Opening Minds Scale for Health Care Providers, PSS: Personal Stigma Scale.

[3] Kelly BJ, Stain HJ, Coleman C, Perkins D, Fragar L, Fuller J, Lewin TJ, Lyle D, Carr VJ, Wilson JM, Beard JR. Mental health and well-being within rural communities: the Australian rural mental health study. *Aust J Rural Health*. 2010;18:16–24.

For peer review only

Supplementary table 3: Quality assessment of the selected studies[1]

Quality of the selected studies		
WEAK	MODERATE	STRONG
Bond et al, 2021	Dimoff et al, 2016	Moll et al, 2018
Kristman et al, 2019	Dobson et al, 2019	
		Svensson and Hansson, 2014
Kubo et al, 2018	Dobson et al, 2021	
Paterson et al, 2021	Eirosa-Orosa et al, 2021	
Quinn et al, 2011	Griffith et al, 2016	
	Hamann et al, 2016	
	Hanisch et al, 2017	
	Moffitt et al, 2014	
	Reavley et al, 2018	
	Shann et al, 2018	
	Szető et al, 2019	
	Tynan et al, 2018	

1: Based on Quality Assessment Tool for Quantitative Studies (QATQS) scale (Ciliska et al, 1998)

For peer review only

Supplementary Table 4: Quality Assessment of the included studies, based on the QATQS

First Author	Selection Bias	Design	Confounders	Blinding	Data Collection Method	Withdrawals and Drop-out	Global Rating
**Bond et al, 2021**	Strong	Moderate	Weak	Moderate	Strong	Weak	**Weak**
**Dimoff et al, 2016**	Weak	Strong	Strong	Strong	Strong	Moderate	**Moderate**
**Dobson et al, 2019**	Moderate	Moderate	Weak	Moderate	Strong	Strong	**Moderate**
**Dobson et al, 2021**	Moderate	Moderate	Weak	Strong	Strong	Strong	**Moderate**
**Eiroa-Orosa et al, 2021**	Moderate	Strong	Weak	Strong	Strong	Moderate	**Moderate**
**Griffith et al, 2016**	Weak	Strong	Strong	Strong	Strong	Moderate	**Moderate**
**Hamann et al, 2016**	Moderate	Moderate	Weak	Moderate	Strong	Strong	**Moderate**
**Hanisch et al, 2017**	Strong	Moderate	Weak	Moderate	Strong	Strong	**Moderate**
**Kristman et al, 2019**	Weak	Moderate	Weak	Moderate	Weak	Moderate	**Weak**
**Kubo et al, 2018**	Strong	Moderate	Weak	Weak	Strong	Strong	**Weak**
**Moffitt et al, 2014**	Moderate	Strong	Strong	Strong	Weak	Strong	**Moderate**
**Moll et al, 2018**	Moderate	Strong	Strong	Strong	Strong	Moderate	**Strong**
**Paterson et al, 2021**	Weak	Moderate	Weak	Strong	Strong	Weak	**Weak**
**Quinn et al, 2011**	Moderate	Moderate	Weak	Weak	Moderate	Strong	**Weak**
**Reavley et al, 2018**	Strong	Strong	Strong	Strong	Strong	Weak	**Moderate**
**Shann et al, 2018**	Weak	Strong	Strong	Strong	Moderate	Moderate	**Moderate**
**Svensson and Hansson, 2014**	Moderate	Strong	Strong	Strong	Strong	Moderate	**Strong**
**Szető et al, 2019**	Strong	Moderate	Weak	Moderate	Strong	Strong	**Moderate**

For peer review only

**Tynan et al, 2018**	Strong	Moderate	Strong	Strong	Weak	Moderate	**Moderate**

For peer review only

Supplementary Figure 1: PRISMA flow chart for the review about workplace interventions against stigma

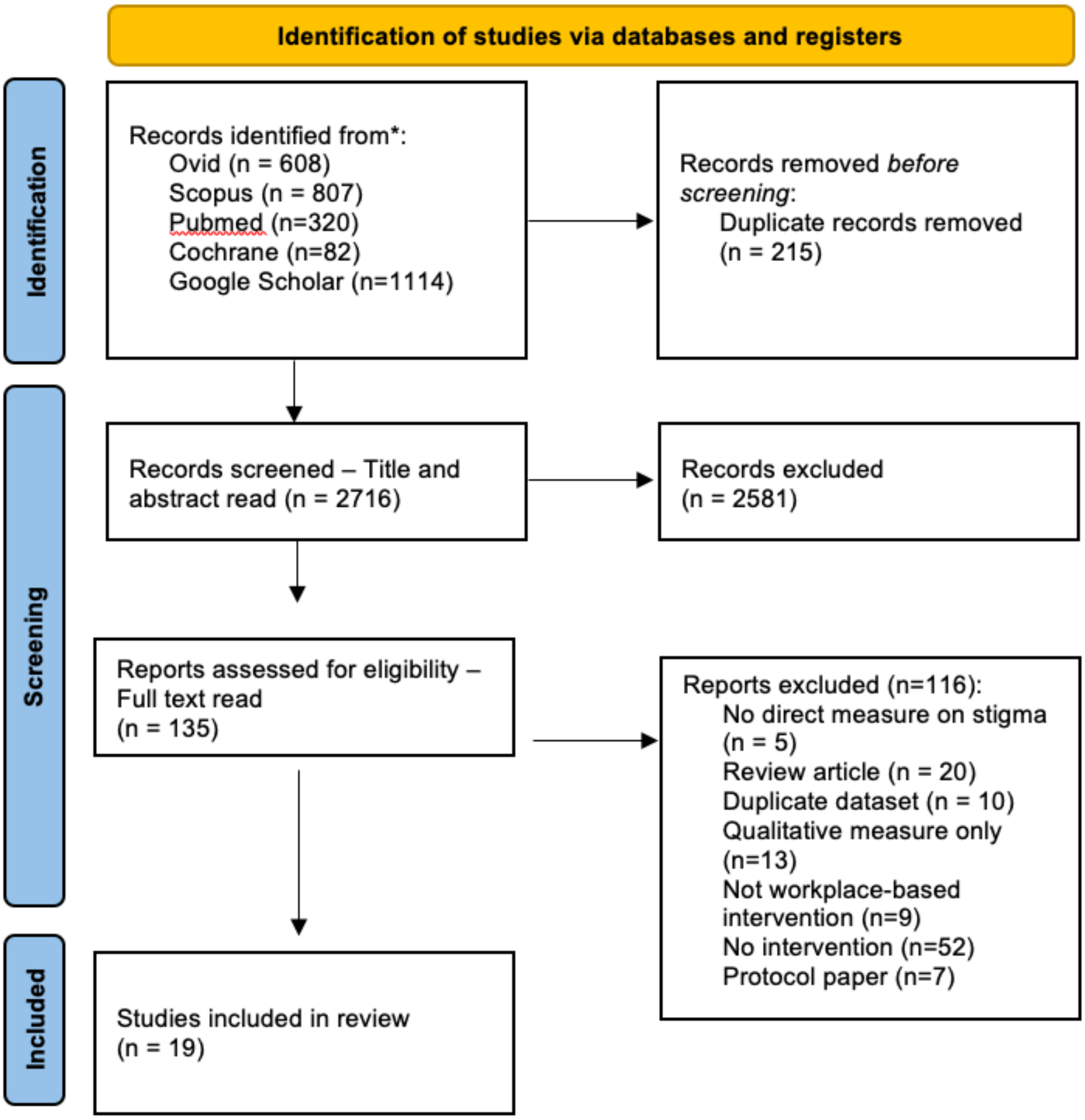

Appendix 1:

The following TI/AB keywords were used:

depress* OR suic* OR anx* OR self-harm OR "mental health" OR discrimination OR exclusion
AND
occupation* or workplace or SME OR job OR "small-sized enterprise*" OR "medium-sized enterprise*" OR "small enterprise*" OR "medium enterprise*" OR "small-sized compan*" OR "medium-sized compan*" OR "small compan*" OR "medium compan*" OR "small-sized business*" OR "medium-sized business*" OR "small business*" OR "medium business*" OR "small-sized organization*" OR "small-sized organisation*" OR "medium-sized organization*" OR "medium-sized organisation*" OR "small organization*" OR "small organisation*" OR "medium organization*" OR "medium organisation*")
AND
anti-stigma OR stigma
AND
reduced OR promot* OR program* OR campaign OR improve* OR intervention OR educat* OR seminar* OR workshop* OR course

For peer review only

PRISMA 2020 Main Checklist

Topic	No.	Item	Location where item is reported
**TITLE**			
**Title**	1	Identify the report as a systematic review.	See Title<br>pp 3.
**ABSTRACT**			
**Abstract**	2	See the PRISMA 2020 for Abstracts checklist	pp4
**INTRODUCTION**			
**Rationale**	3	Describe the rationale for the review in the context of existing knowledge.	See Introduction<br>pp5
**Objectives**	4	Provide an explicit statement of the objective(s) or question(s) the review addresses.	See Introduction<br>pp5
**METHODS**			
**Eligibility criteria**	5	Specify the inclusion and exclusion criteria for the review and how studies were grouped for the syntheses.	See study selection section<br>pp6
**Information sources**	6	Specify all databases, registers, websites, organisations, reference lists and other sources searched or consulted to identify studies. Specify the date when each source was last searched or consulted.	See included studies section<br>pp7
**Search strategy**	7	Present the full search strategies for all databases, registers and websites, including any filters and limits used.	See appendix 1 and search strategy section<br>pp7 and pp37
**Selection process**	8	Specify the methods used to decide whether a study met the inclusion criteria of the review, including how many reviewers screened each record and each report retrieved, whether they worked independently, and if applicable, details of automation tools used in the process.	See study selection section<br>pp6
**Data collection process**	9	Specify the methods used to collect data from reports, including how many reviewers collected data from each report, whether they worked independently, any processes for obtaining or confirming data from study investigators, and if applicable, details of automation tools used in the process.	See review procedure section<br>pp6

Topic	No.	Item	Location where item is reported
**Data items**	10a	List and define all outcomes for which data were sought. Specify whether all results that were compatible with each outcome domain in each study were sought (e.g. for all measures, time points, analyses), and if not, the methods used to decide which results to collect.	See data extraction section pp 7
	10b	List and define all other variables for which data were sought (e.g. participant and intervention characteristics, funding sources). Describe any assumptions made about any missing or unclear information.	See data extraction section pp 7
**Study risk of bias assessment**	11	Specify the methods used to assess risk of bias in the included studies, including details of the tool(s) used, how many reviewers assessed each study and whether they worked independently, and if applicable, details of automation tools used in the process.	Supplementary Table 3. pp 29
**Effect measures**	12	Specify for each outcome the effect measure(s) (e.g. risk ratio, mean difference) used in the synthesis or presentation of results.	n/a
**Synthesis methods**	13a	Describe the processes used to decide which studies were eligible for each synthesis (e.g. tabulating the study intervention characteristics and comparing against the planned groups for each synthesis (item 5)).	n/a
	13b	Describe any methods required to prepare the data for presentation or synthesis, such as handling of missing summary statistics, or data conversions.	n/a
	13c	Describe any methods used to tabulate or visually display results of individual studies and syntheses.	n/a
	13d	Describe any methods used to synthesize results and provide a rationale for the choice(s). If meta-analysis was performed, describe the model(s), method(s) to identify the presence and extent of statistical heterogeneity, and software package(s) used.	n/a
	13e	Describe any methods used to explore possible causes of heterogeneity among study results (e.g. subgroup analysis, meta-regression).	n/a
	13f	Describe any sensitivity analyses conducted to assess robustness of the synthesized results.	n/a

For peer review only - http://bmjopen.bmj.com/site/about/guidelines.xhtml

Topic	No.	Item	Location where item is reported
**Reporting bias assessment**	14	Describe any methods used to assess risk of bias due to missing results in a synthesis (arising from reporting biases).	See Table 4. pp 31
**Certainty assessment**	15	Describe any methods used to assess certainty (or confidence) in the body of evidence for an outcome.	n/a
**RESULTS**			
**Study selection**	16a	Describe the results of the search and selection process, from the number of records identified in the search to the number of studies included in the review, ideally using a flow diagram.	See Result section pp8
	16b	Cite studies that might appear to meet the inclusion criteria, but which were excluded, and explain why they were excluded.	See study selection section pp6
**Study characteristics**	17	Cite each included study and present its characteristics.	Supplementary Table 1 and 2. pp 19 and 24.
**Risk of bias in studies**	18	Present assessments of risk of bias for each included study.	Supplementary Table 4. pp 31.
**Results of individual studies**	19	For all outcomes, present, for each study: (a) summary statistics for each group (where appropriate) and (b) an effect estimate and its precision (e.g. confidence/credible interval), ideally using structured tables or plots.	Supplementary Table 2. pp 24.
**Results of syntheses**	20a	For each synthesis, briefly summarise the characteristics and risk of bias among contributing studies.	n/a
	20b	Present results of all statistical syntheses conducted. If meta-analysis was done, present for each the summary estimate and its precision (e.g. confidence/credible interval) and measures of statistical heterogeneity. If comparing groups, describe the direction of the effect.	n/a
	20c	Present results of all investigations of possible causes of heterogeneity among study results.	n/a
	20d	Present results of all sensitivity analyses conducted to assess the robustness of the synthesized results.	n/a

Topic	No.	Item	Location where item is reported
**Reporting biases**	21	Present assessments of risk of bias due to missing results (arising from reporting biases) for each synthesis assessed.	n/a
**Certainty of evidence**	22	Present assessments of certainty (or confidence) in the body of evidence for each outcome assessed.	Supplementary Table 2. pp 24.
**DISCUSSION**			
**Discussion**	23a	Provide a general interpretation of the results in the context of other evidence.	See Discussion section pp 11.
	23b	Discuss any limitations of the evidence included in the review.	See limitation section pp 13
	23c	Discuss any limitations of the review processes used.	See limitation section pp 13
	23d	Discuss implications of the results for practice, policy, and future research.	See Conclusions section pp13
**OTHER INFORMATION**			
**Registration and protocol**	24a	Provide registration information for the review, including register name and registration number, or state that the review was not registered.	See data extraction section pp 7
	24b	Indicate where the review protocol can be accessed, or state that a protocol was not prepared.	See review procedure section pp 6
	24c	Describe and explain any amendments to information provided at registration or in the protocol.	n/a
**Support**	25	Describe sources of financial or non-financial support for the review, and the role of the funders or sponsors in the review.	See Funding section pp 14

For peer review only - http://bmjopen.bmj.com/site/about/guidelines.xhtml

Topic	No.	Item	Location where item is reported
**Competing interests**	26	Declare any competing interests of review authors.	See Competing interest interest section<br>pp 14
**Availability of data, code and other materials**	27	Report which of the following are publicly available and where they can be found: template data collection forms; data extracted from included studies; data used for all analyses; analytic code; any other materials used in the review.	n/A

For peer review only

PRIMSA Abstract Checklist

Topic	No.	Item	Reported?
**TITLE**			
**Title**	1	Identify the report as a systematic review.	Yes
**BACKGROUND**			
**Objectives**	2	Provide an explicit statement of the main objective(s) or question(s) the review addresses.	Yes
**METHODS**			
**Eligibility criteria**	3	Specify the inclusion and exclusion criteria for the review.	Yes
**Information sources**	4	Specify the information sources (e.g. databases, registers) used to identify studies and the date when each was last searched.	Yes
**Risk of bias**	5	Specify the methods used to assess risk of bias in the included studies.	Yes
**Synthesis of results**	6	Specify the methods used to present and synthesize results.	Yes
**RESULTS**			
**Included studies**	7	Give the total number of included studies and participants and summarise relevant characteristics of studies.	Yes
**Synthesis of results**	8	Present results for main outcomes, preferably indicating the number of included studies and participants for each. If meta-analysis was done, report the summary estimate and confidence/credible interval. If comparing groups, indicate the direction of the effect (i.e. which group is favoured).	Yes
**DISCUSSION**			
**Limitations of evidence**	9	Provide a brief summary of the limitations of the evidence included in the review (e.g. study risk of bias, inconsistency and imprecision).	Yes
**Interpretation**	10	Provide a general interpretation of the results and important implications.	Yes
**OTHER**			
**Funding**	11	Specify the primary source of funding for the review.	Yes
**Registration**	12	Provide the register name and registration number.	Yes

For peer review only - http://bmjopen.bmj.com/site/about/guidelines.xhtml

From: Page MJ, McKenzie JE, Bossuyt PM, Boutron I, Hoffmann TC, Mulrow CD, et al. The PRISMA 2020 statement: an updated guideline for reporting systematic reviews. MetaArXiv. 2020, September 14. DOI: 10.31222/osf.io/v7gm2. For more information, visit: www.prisma-statement.org

For peer review only

For peer review only - http://bmjopen.bmj.com/site/about/guidelines.xhtml

BMJ Open

Evidence for the effectiveness of interventions to reduce mental health related stigma in the workplace: A Systematic Review

Journal:	*BMJ Open*
Manuscript ID	bmjopen-2022-067126.R1
Article Type:	Original research
Date Submitted by the Author:	24-Nov-2022
Complete List of Authors:	Ditta Tóth, Mónika; Semmelweis University, Institute of Behavioural Sciences<br>Ihionvien, Sarah; Semmelweis University, Institute of Behavioural Sciences<br>Leduc, Caleb ; University College Cork, School of Public Health<br>Aust, Birgit; National Research Centre for the Working Environment<br>Amann, Benedikt; Hospital del Mar Institute for Medical Research, Mental Health Group; Univ. Pompeu Fabra<br>Cresswell-Smith, Johanna; THL<br>Reich, Hanna; German Foundation for Research and Education on Depression; German Foundation for Research and Education on Depression<br>Cully, Grace; University College Cork School of Public Health<br>Sanches, Sarita; Phrenos Center of Expertise; Altrecht Foundation for Mental Health Care<br>Fanaj, Naim; Mental Health Center, ; Mental Health Center Prizren<br>Qirjako, Gentiana; University of Medicine, Department of Public Health<br>Tsantila, Fotini; KU Leuven, LUCAS, Centre for Care Research and Consultancy<br>Ross, Victoria ; Australian Institute for Suicide Research and Prevention<br>Mathieu, Sharna; Australian Institute for Suicide Research and Prevention<br>Pashoja, Arlinda; London School of Hygiene and Tropical Medicine Faculty of Public Health and Policy, Faculty of Epidemiology and Population Health<br>Arensman , Ella ; University College Cork, School of Public Health; National Suicide Research Foundation<br>Purebl, György; Semmelweis University, Institute of Behavioural Sciences
**Primary Subject Heading**:	Occupational and environmental medicine
Secondary Subject Heading:	Public health
Keywords:	MENTAL HEALTH, OCCUPATIONAL & INDUSTRIAL MEDICINE, Depression & mood disorders < PSYCHIATRY

SCHOLARONE™
Manuscripts

I, the Submitting Author has the right to grant and does grant on behalf of all authors of the Work (as defined in the below author licence), an exclusive licence and/or a non-exclusive licence for contributions from authors who are: i) UK Crown employees; ii) where BMJ has agreed a CC-BY licence shall apply, and/or iii) in accordance with the terms applicable for US Federal Government officers or employees acting as part of their official duties; on a worldwide, perpetual, irrevocable, royalty-free basis to BMJ Publishing Group Ltd ("BMJ") its licensees and where the relevant Journal is co-owned by BMJ to the co-owners of the Journal, to publish the Work in this journal and any other BMJ products and to exploit all rights, as set out in our licence.

The Submitting Author accepts and understands that any supply made under these terms is made by BMJ to the Submitting Author unless you are acting as an employee on behalf of your employer or a postgraduate student of an affiliated institution which is paying any applicable article publishing charge ("APC") for Open Access articles. Where the Submitting Author wishes to make the Work available on an Open Access basis (and intends to pay the relevant APC), the terms of reuse of such Open Access shall be governed by a Creative Commons licence – details of these licences and which Creative Commons licence will apply to this Work are set out in our licence referred to above.

Other than as permitted in any relevant BMJ Author's Self Archiving Policies, I confirm this Work has not been accepted for publication elsewhere, is not being considered for publication elsewhere and does not duplicate material already published. I confirm all authors consent to publication of this Work and authorise the granting of this licence.

For peer review only

Evidence for the effectiveness of interventions to reduce mental health related stigma in the workplace: A Systematic Review

Mónika Ditta Tóth[1], Sarah Ihionvien[1], Caleb Leduc[2,3], Birgit Aust[4], Benedikt L. Amann[5,6,7,8,9] Johanna Cresswell-Smith[10], Hanna Reich[11,12], Grace Cully[2], Sarita Sanches[13,14], Naim Fanaj[15], Gentiana Qirjako[16], Fotini Tsantila[17], Victoria Ross[18], Sharna Mathieu[18], Arlinda Cerga Pashoja[19], Ella Arensman[2,3,18], György Purebl[1]

1: Institute of Behavioural Sciences, Semmelweis University, Budapest, Hungary
2: School of Public Health, University College Cork, Cork, Ireland
3: National Suicide Research Foundation, Cork, Ireland
4: National Research Centre for the Working Environment, Copenhagen, Denmark
5: Centre Fòrum Research Unit, Institute of Neuropsychiatry and Addiction (INAD), Parc de Salut Mar, Hospital del Mar Medical Research Institute (IMIM), Barcelona, CIBERSAM, Spain
6: Univ. Pompeu Fabra, Barcelona, Spain
7: Department of Psychiatry and Psychotherapy, Klinikum der Universität München, Munich, Germany
8: Centro de Investigación Biomédica en Red de Salud Mental, Instituto de Salud Carlos III, Madrid, España.
9. Department for Psychiatry and Psychotherapy, Hospital of the Ludwig-Maximilians-University Munich, Germany
[10]Finnish Institute for Health and Welfare (THL)
11: German Depression Foundation, Leipzig, Germany
12: Depression Research Centre of the German Depression Foundation, Department of Psychiatry, Psychosomatic Medicine and Psychotherapy, University Hospital, Goethe University, Frankfurt am Main, Germany
13: Phrenos Center of Expertise for severe mental illness, Utrecht, The Netherlands
14: Altrecht Mental Health Care, Utrecht, The Netherlands
15: Mental Health Center Prizren, Kosovo & College of Medical Sciences Rezonanca Prishtina, Kosovo
16: Department of Public Health, University of Medicine, Tirana, Albania
17: LUCAS, Centre for Care Research and Consultancy, KU Leuven, 3000 Leuven, Belgium;
18: Australian Institute for Suicide Research and Prevention & WHO Collaborating Centre for Research and Training in Suicide Prevention, School of Applied Psychology, Griffith University, Brisbane, Australia
19: Faculty of Epidemiology and Population Health. London School of Hygiene and Tropical Medicine, UK, London

Corresponding author:

Dr. Mónika Ditta Tóth
E-mail: tmonika85@gmail.com
Address: 1089 Budapest, Nagyvárad tér 4. Hungary
Telephone: +36303168621

Keywords:Mental health, stigma, workplace

Word count: 4655

For peer review only

Abstract

Objective Increasing access to mental health support is a key factor for treating mental disorders, however, important barriers complicate help-seeking, among them, mental health related stigma being most prominent. We aimed to systematically review the current evidence for interventions focusing on reducing stigma related to mental health problems in Small and Medium Enterprises (SMEs).

Design Systematic review with a focus on interventions targeting mental health related stigma in the workplace in accordance with PRISMA guidelines. The methodological quality of included article was assessed using the QATQS scale.

Data sources PubMed, Ovid Medline, PsycINFO, Scopus, and Cochrane databases and Google Scholar were searched from January 2010 until November 2022.

Eligibility criteria for selecting studies We included experimental or quasi-experimental studies about workplace interventions aiming to reduce stigma, where the outcomes were measured in terms of stigmatization against depression, anxiety and/or other mental health problems.

Data extraction and synthesis Records were screened by two independent reviewers after inspecting titles and abstracts and a full-text read of the articles to assess whether they met inclusion criteria. The results were synthezised narratively.

Results. We identified 22 intervention studies, three with high quality, 13 with moderate quality and 6 with weak quality. Only two studies included SMEs, but no study focused on SMEs exclusively . The mode of delivery of the intervention was face-to face in 15 studies, online in four studies, and mixed in three studies. We found a significant reduction in stigmatizing attitudes in almost all studies (20/22), using ten different instruments/scales. Effects seemed to be independet of company size. Online interventions were found to be shorter, but seemed to be as effective as face-to-face interventions.

Conclusions. Although we did not find interventions focusing exclusively on SMEs, it is likely that anti-stigma interventions also will work in smaller workplaces.

Strengths and limitations of this study

- The present systematic review was based on a comprehensive search identifying 22 studies providing an important update since a similar review published in 2016
- The methodological quality of the identified studies was assessed by two independent reviewers using the Quality Assessment Tool for Quantitative Studies (QATQS) scale
- Given the diverse study designs and outcome measures, no meta-analysis was possible to conduct.
- Only studies with quantitative measurement were included in this review, however qualitative studies could provide important additional information, especially about the mechanisms leading to changes in stigma attitudes .

For peer review only

- Because of our search strategy and inclusion criteria - we cannot distinguish between different types of stigma-related changes: knowledge, beliefs and behaviours.

Introduction

Mental disorders can have significant consequences, not only on the individual level, but also on a societal and economic level. In the context of the workplace[1,2], poor mental health has been linked with absenteeism and presenteeism[3, 4, 5] leading to decreased workplace performance, productivity, and increased risk of unemployment[6, 7]. Depression and anxiety are the two most common mental disorders globally, and are therefore also most likely to impact work performance and productivity[8].

Increasing access to mental health support is a key factor for treating mental disorders. Research highlights several important barriers which complicate help-seeking, with mental health related stigma being the most prominent[9]. Stigma can be defined as the convergence of several interrelated components, such as labeling, stereotyping, separation, status loss, and discrimination which occur together[10]. This includes perceived stigma (also known as social stigma) relating to an individual's perception of what others think and feel, and personal stigma (also known as self-stigma) reflecting individual thoughts and attitudes restricting openness about mental health difficulties, increasing risk of social exclusion, and limiting help seeking behavior[11, 12]. In a nationwide US study, over 90% of first responders found stigma as a main barrier to seeking help for themselves[9]. International evidence indicates that experiences of stigma and discrimination lead to decreased use of mental-health related interventions, including workplace-based mental health promotion programmes[13, 14, 15]. Mental health-related stigma can also lead to the breakdown of social connections including avoidance, rejection, and a perception of reduced competence[16]. As a consequence, the person involved may experience lack of career development, reduction of responsibilities, inequity in workplace policies, and exclusion from work integration and social activities. Stigma has also been found to increase the risk of unemployment, job uncertainty, and reduced the likelihood of being hired [17].

Addressing mental health related stigma is a central component of LaMontagne's [18] model for workplace mental health, which integrates preventing harm and reducing risk factors, promoting the positive aspects of work, and management of mental illness. Investing in mental health in the workplace via mental health promotion actions can not only improve mental health on an individual level, but also increase economic productivity[19, 20, 21]. Several workplace-based mental health promotion programmes have been implemented in the EU, with the majority of these being conducted in large companies. This means that interventions are only reaching a small proportion of all employees as the majority (99%) of EU-based workplaces represent Small or Medium size Enterprises (SMEs)[22]. Despite proportionally more people being employed by SMEs in comparison to larger companies, SMEs often lack the financial and/or human resources (HR) support for mental health promotion. Although face-to-face interventions seem to be more effective, research shows that online interventions can be time- and cost-effective, and also easily implementable which can be favourable for small enterprises with presumably limited budgets to implement mental health promotion activities[23].

Although research has shown that stigma can lead to a number of negative consequences and is a barrier for workplace mental health promotion, more insight is required into how best to

For peer review only

reduce stigma. A number of intervention studies investigating the effects of anti-stigma initiatives have been conducted during the last 10-20 years, and so far only one systematic review has been published[24]. This review identified 16 intervention studies targeting stigma of mental illness at the workplace. The review included research published between 2004 and 2014 and found support for anti-stigma interventions leading to improved employee knowledge and supportive behaviour towards people with mental-health problems. They concluded that while the majority of interventions demonstrated a positive effect on employees' attitudes, there remained significant need for improved methodological quality in future evaluations. Specifically, selection bias might have contributed to the positive effects. In particular, one of the main findings indicated that the majority of the interventions were conducted with more highly educated supervisors or in job groups, with more highly educated employees and in the public sector. This reduces the generalizability to most workplaces in other diverse sectors with less educated workers. Consistent with workplace mental health research in general, most of these studies were also conducted in larger organizations, and therefore not providing any knowledge about interventions designed to reduce stigma in SMEs. The currently ongoing intervention project Mental Health Promotion and Intervention in Occupational Settings (MENTUPP Project) aims to contribute to knowledge in this area. A comprehensive online intervention has been developed and is currently being tested in a number of SMEs across European countries and Australia[25]. This review has been conducted as part of the MENTUPP Project to enhance its evidence base.

Therefore, the main aim of this paper was to systematically review the current evidence for interventions focusing on reducing stigma related to mental health problems in SMEs in various sectors. A secondary aim of the review was to investigate the mode of delivery and intensity/duration of interventions.

Methods

Review procedure

A systematic literature search was conducted with a focus on interventions targeting mental health related stigma in the workplace. The review was conducted in accordance with the PRISMA guideline process[26]. Peer-reviewed articles about workplace-based anti-stigma interventions were searched from January 2010 until 14th July 2021 via PubMed, Ovid Medline, PsycINFO, Scopus and Cochrane databases. An additional Google Scholar search was conducted. All results from the database search were uploaded to Covidence (www.covidence.org), an online tool for managing and streamlining systematic reviews.

Study selection

The systematic review was conducted addressing the following inclusion criteria: 1) the sample included employees and/or owners/managers; 2) the intervention at the workplace was aimed to reduce stigma; 3) the outcomes were measured in terms of stigmatization against depression, anxiety and/or other mental health problems; 4) studies had an experimental or quasi-experimental design (including quantitative data); 5) the studies were published in English; 6) the intervention was delivered through the workplace; and 7) the studies were published between January 2010 and July 2021.
Studies were excluded based on the following criteria: 1) no evaluation of the intervention; 2) only qualitative evaluation (e.g. interview or focus group); or 3) no direct measure on stigma

For peer review only - http://bmjopen.bmj.com/site/about/guidelines.xhtml

(studies with indirect measures of stigma, such as knowledge of mental health, or attitudes towards mentally ill patients, were excluded).

After duplicates were removed, the records were screened by two independent reviewers (GyP, SI) following a two-stage procedure: 1. Inspecting titles and abstracts of the studies, and 2. A full-text read of the articles to assess whether they met inclusion criteria. In the case of disagreement, a consensus was made together with a third researcher (MDT; first author of the study).

Search strategy

The search string was developed by GYP and MDT, reviewed by IS and CL, and subsequently reviewed by a subject librarian at Semmelweis University Hungary (See search keywords in Appendix 1). Terms related to the following themes were used: mental health related terms AND workplace related terms AND stigma-related terms AND intervention related terms.

Included studies

Supplementary figure 1. displays the PRISMA flow diagram which shows the decision points during the screening process.

(SUPPLEMENTARY FIGURE 1)

The PubMed, Ovid Medline, PsycINFO, Scopus and Cochrane databases and Google Scholar were searched resulting in initial identification of 3479 articles. After removal of duplicates (n=221) title screening and abstract review was conducted for 3258 articles, of which 154 were retained for full-text screening, and 23 met criteria for inclusion. However two articles (Reavley 2018 and 2021) reported about the same intervention study, which means that 22 intervention studies were identified.

Data extraction

Data extraction by two co-authors for the articles after full-text review included the following and was independently cross-checked by a third reviewer (MDT): (1) Author and year; (2) study design; (3) number of participants at baseline and follow up; (4) gender of participants (5) Target group (6) Sector and Size of organization (7) intervention; (8) intervention intensity; (9) country (10) (Supplementary Table, 1) outcome measure on stigma; (11) evaluation timepoints; (12) main findings (Supplementary Table 2).

The review was conducted according to PRISMA guidelines[26] and registered with PROSPERO: ID: CRD42020191307.

Quality assessment

For peer review only

The methodological quality of each included article was assessed using the Quality Assessment Tool for Quantitative Studies (QATQS) scale[27], based on the following aspects rated from weak to strong: selection bias, design, confounders, blinding, data collection method and drop-out. The global rating was high, in case of 'no weak rating', moderate in case of one 'weak rating', and weak in case of 'two or more weak ratings'. Quality assessment was finalized after two independent reviews by the first and second author of this review, followed by a consensus meeting together with a third independent reviewer GYP.

Patient and Public Involvement

No patient involved.

Results

Study Characteristics

Of the 22 included intervention studies, seven were conducted in Canada, six in Australia, four in Great Britain, two in Germany and one each in Sweden, Spain and Japan. Nine studies used a Randomized Controlled Trial (RCT) study design and the remaining 13 used a quasi-experimental design. An overview of the studies is presented in supplementary table 1 and 2.

(SUPPLEMENTARY TABLE 1)

(SUPPLEMENTARY TABLE 2)

Sector and size of organization

A total of 22 interventions were used by the included studies, most of which (12/22) were conducted in public sector organizations, or in a mixture of public and private sector workplaces (4/22). Only four studies focused solely on private sector companies, and no sector-specific information was provided in two of the studies. The interventions enrolled different professional groups in varying positions including healthcare workers (2 studies), first responders (4), public servants (2), maintenance staff (2), governmental employees (2), housing association (1), managers, leaders (8), hospitality industry (1).

Six studies provided information on the size of the organizations, the four studies in the private sector enrolled large enterprises with more than 250 employees. Two interventions enrolled a mixture of small, medium and large organizations. No intervention study specifically focused on SMEs.

Quality assessment of the studies

The assessed methodological quality of the included studies varied from weak to strong, with three considered to be of high quality. Almost two thirds of papers (13/22) were assessed as having moderate quality, most lacking a control group design. Six articles were appraised as weak, a rating driven primarily from low agreement rate and/or high dropout rate (supplementary table 3).

(SUPPLEMENTARY TABLE 3)

The detailed evaluation criteria of the QATQS scale are presented in supplementary table 4.

For peer review only

(SUPPLEMENTARY TABLE 4)

Interventions

Overall, ten interventions used previously developed standardized interventions, including the Mental Health First Aid program, Psychological First Aid, Applied Suicide Intervention Skills Training, Beyond Blue or Mental Health-Guru, with other interventions being designed or modified to fit a workplace-based context. Twelve interventions used non-standardized mental health approaches. In terms of implementation, four interventions included in the studies were delivered online, 15 delivered in person, and three were blended interventions (delivered both online and face to face). All programmes used multimodal approaches, which included multiple intervention techniques such as psychoeducation, interactive skills training exercises, and case vignettes/videos of experts with lived experience. Some of the interventions contained specific leadership-focused elements. The most frequent topics were: education about the features and symptoms of mental disorders (special focus on depression and anxiety), warning signs of mental disorders, crisis and suicidal risk and its management, importance of mental health issues in the workplace, and communication strategies for supporting employees with mental health problems.

As a general result we found a significant reduction in stigmatizing attitudes in almost all studies (20/22), using ten different instruments/scales. A detailed overview of study characteristics is presented in Supplementary Table 1 and the main findings of each study are presented in Supplementary Table 2.

Mode of delivery

In the next section we will shortly describe some main features of the 22 studies. First, we present the online interventions, then the face-to-face interventions and finally the blended interventions. Within each category we begin with presenting studies with a RCT design followed by studies with a quasi-experimental or other study designs.

Online interventions

Four out of the 22 studies delivered the intervention in an online format[28 - 31]. Out of the four studies, three found significant positive effects on stigmatizing attitudes, while one intervention did not find a positive effect after the intervention[31]. The average length of these online interventions was 146 minutes, the shortest being 30-45 minutes and the longest six hours. The positive effects were maintained at three[29] and six months follow up[28, 30].

RCT design studies

Griffiths et al investigated the effectiveness of a 1-hour long online mental health programme for employees of governmental organizations (N=507) [28]. Significant reduction measured by the personal subscales of The Depression and Generalized Anxiety Stigma Scales[32, 33] was found post intervention and 6 months follow-up. **Shann et al** delivered an online leadership intervention (N=311) [30]. Even a short, 30-45 mins duration intervention resulted in a significant reduction in stigma scores even at 6-month follow up, which was measured by a 12-item Managerial Stigma Toward Employee Depression Scale[34].

Studies with non RCT design

For peer review only

Paterson et al delivered a 6-hour long online workplace intervention (N=134) [31]. No significant difference in pre and post measures stigma scores between intervention and control group was found, and the methodological quality was rated as weak. The adopted version of King's stigma Scale was used[35]. **Hanisch** et al. delivered a 2-hour digital training for managers (N=48)[29]. The intervention resulted in significant reduction regarding stigmatization toward people with mental health problems, but no control group was enrolled. The Opening Minds Scale for Workplace Attitudes was used post-training and at 3 months follow-up[35].

Face-to-face interventions

Most of the studies used a face-to-face approach (15 out of22). The average length of these interventions was 10.1 hours (=606 minutes), the shortest being two hours and the longest 16 hours interventions. Only one intervention did not find a significant positive effect on stigmatizing attitudes[36], and one revealed rebound effect at 3 months follow up[37]. Two further studies did not have a follow-up measurement [38, 39]. The length of the follow up varied between 1 month to 2 years.

Studies with RCT design

Six studies used RCT designs, one rated as a methodologically strong study: **Svensson and Hansson**[40] conducted a 12-hour long training for public sector employees (N=199). A vignette version of the Depression Personal and Perceived Stigma scale[32] showed significant reduction in personal stigma towards people with depression after 6 months and even at 2 years follow up, but no significant changes were found in the control group. Similarly, the other four studies[41, 42, 43, 37] found significant reduction in stigmatizing attitudes in their intervention group post training, and 1-3 months follow-up, but no significant changes were found in the control groups. The effects of 3 to 7.5 hours face to face trainings were measured by the modified version of the Depression Stigma Personal subscale[32], the Opening Minds Scale for Workplace Attitude[35], the Opening Minds Stigma Scale for Health Care Providers[44] and the Mental Health Knowledge Scale [43]. Fire service line managers (N=106) were randomly assigned to either a 2-day or 12 hour long training group or a control group (1 hour leaflet session)[39]. The locally developed Knowledge and Efficacy about Mental Health Problems scale revealed statistically significant improvements in stigma on mental health pre- and post-training in both training settings, but not in the control group.

Studies with non-RCT design

Five out of six *weak methodological quality* interventions were performed a face-to face non-RCT design.

Bond et al. [45] delivered a 4-hour course for employees in support services (N=284). Significant reduction was found on stigmatizing items measured by an adapted version of Depression Stigma Scale[12] after the course and 6-month follow up. **Kubo** et al[46] delivered a 2-hour long education program (N=91). Right after the intervention, the Japanese version of the Links Perceived Devaluation-Discrimination Scale[10] showed a significant decrease in negative attitudes towards mental health problems, but this difference was not maintained after 1 month. Although there was a long-term (2 years) effect in perceived mental health stigma in **Kristman's et al**[47] 2 year-long quasi-experimental study (N=89), the methodological quality

For peer review only

of the study was assessed as weak. **Quinn et al**[48]conducted a 6-hour long training course for telecommunication workers (N=101). Relevant questions gathered from the Scottish Public Attitudes Survey[49] revealed a significant decrease in stigmatizing attitudes between pre and post intervention, however the methodology was rated as weak. **Stelnicki** et al [50] conducted a 16-hour long program for public safety personel (N=136) which resulted in significant decrease in stigma scores measered by the Opening Minds Scale for Workplace Attitude [35].

Five other face-to-face studies were rated as having *moderate methodological quality*: **Dobson** et al[51] (N=1292) and **Szető et al** (N=5598) investigated the effects of a 4- and 8-hour long stigma reduction program for frontline workers and managers[52] . In both studies, the Opening Minds Scale for Workplace Attitudes[35] showed a significant reduction in stigma for the total scale and all the subscales between pre- and post- intervention and 3-month follow up in both groups. In their longitudinal cohort study, **Hamann** et al [38] delivered a 1-1.5-day long face-to-face educational workshop for leaders and HR department employees (N=580). Post-intervention, the Depression Stigma Personal Subscale[32] showed a significant decrease, but no follow-up measure was performed. On the other hand, reduction in stigma was not significant in a 1-hour training followed by an 4-hour gate-keeper training for Australian Mates in Mining co-workers (N=1275) and 117 supervisors[36]. Mental health stigma was measured by Perceived Stigma Scale[53].

Blended studies

All of the blended design studies used randomized designs. In a study by **Moll et al** with strong methodological quality, mental health literacy training was delivered to healthcare workers (N=192) in either face-to-face or blended setting [54]. Both interventions resulted in a significant reduction of stigmatizing beliefs, but a longer effect was seen by the blended intervention at 6-month follow up, which was measured by the Opening Minds Scale for Health Care Providers[44]. In a study by **Reavley et al** 608 public sector employees were randomized into different interventions: two MHFA and PFA online courses and a blended MHFA one[55, 56]. Significant reduction in stigma scores were found in each intervention groups post training and 1 year follow up and the Personal Stigma Scale[12] showed no significant difference between online and blended courses. Lam et al’s [57] 3-months long study delivered an online Mental Health First Aid training combined with face-to face sessions for various large enterprise employers (N=456). The strong methodological quality study resulted in a significant reduction of stigma scores post training and at 3-months follow up.

Discussion

The main aim of this systematic review was to identify and evaluate the effectiveness of different workplace-based anti-stigma interventions, focusing on reducing stigmatizing attitudes and discrimination of people with mental illness. The review included interventions that were delivered to employees and employers. A specific focus was placed on Small and Medium Enterprises.

Twenty-two articles met the inclusion criteria and we found an overall positive effect for most of the interventions irrespective of the mode of delivery. Three of the four studies using online interventions found positive effects. Among the fifteen face-to-face interventions, only one study did not find an effect, although a few studies only found short term effects. This finding appears to indicate that online anti-stigma interventions can be just as effective as face-to-face interventions. Similarly, a study comparing training for managers to improve their confidence

For peer review only

in supporting the mental health of their employees found that both the online and face-to-face version to be effective[23]. As for the intensity of the intervention, we can conclude that the average length of online interventions was substantially shorter compared to those delivered face-to-face (146 min vs 606 min on average).

The finding that online interventions might be just as effective as face-to-face interventions was also confirmed by two further randomized controlled studies identified in this review. Reavley et al[55, 56] found no significant difference between the effectiveness of blended and purely online interventions on stigmatizing attitudes, and a longer lasting positive effect was found in a blended intervention compared to its face-to-face version in another study[54]. These results underline the possible benefits of online interventions over the conventional face-to-face approaches: online interventions are shorter, need no presence of the professionals/trainers, and they have particular potential for the workplace as they can be tailored to participant or workplace needs (i.e., can be used anytime during the day), which may also have favourable cost implications. These features make them especially attractive for SMEs as they typically have fewer resources for implementing workplace mental health interventions. Online interventions can also be beneficial during public health emergencies (such as the Covid-19 pandemic) when face-to-face contact is reduced or not possible.

We can conclude that the quality of the interventions has improved since Hanisch et al.'s review[24], having only three overlapping studies with this previous review (39, 40, 47). We identified studies with larger sample size and longer lasting effects. Our review also confirms the findings of the previous review with more studies with higher methodological quality. However, in this review the majority of the identified studies did not have a control group and the drop-out rate in some studies was high. Only two of the twenty-two studies were rated to have strong methodological quality. The majority of the programmes used a multitude of intervention techniques targeting both employees and leaders, which may have made the intervention more effective, but produces difficulties in terms of identifying the most effective elements for stigma-reduction.

With regard to evaluation aspects, 17 studies included follow-up measurements after the intervention, with the duration varying from 1 month to 2 years. Most of the studies used a 1 to 6-month follow up, only two programs followed their participants for 2 years, and both found that the effects were maintained. A few studies however, reported only short-term effects. It remains unclear why some interventions demonstrate long-term effects while other studies only achieved short term effects. More studies with longer follow-up time and more studies with more details about the content of the intervention are needed to investigate this further.

Despite the overall positive outcomes on stigmatizing attitudes by the reviewed studies, it would be important to know if employees actually experience a reduction in exposure to mental health related stigma from their colleagues and managers following the interventions. Measurement tools assess changes in attitudes that do not always translate into differences in behavior and other measures should more frequently be applied in these studies, such as the willingness to seek or offer help.

One of the 2 studies which did not find a significant reduction in stigmatizing attitudes after the intervention, investigated the effects of a 6-hour long online training programme[31]. Authors concluded that the stigma questionnaire[33] used in their evaluation may not have been sensitive enough to capture improvement in mental health related stigma in the workplace context. Similarly, a non-validated stigma-measuring scale could be the reason of another intervention which seems to have no significant reduction in stigma scores[36].

For peer review only

Although our primary aim was to review changes in mental health related stigma, other results are also noteworthy. For example, some interventions were also found to contribute to increased mental health literacy[54, 41] and intention to seek help[28]. Increased resilience [51, 52] and help-seeking behavior[28, 54] were also observed, confirming previous findings by Hanisch et al[23].

Workplace-based mental health stigma-reduction programs appear to have very similar key objectives and approaches, although we noted a tendency to use different evaluation approaches using different scales. The use of appropriate, psychometrically sound scales to assess stigma is crucial and facilitates comparison of findings. Both of the interventions [31, 36] with no significant reductions in stigma scores applied scales that may not have been sensitive enough in workplace settings. Moreover, some researchers used semi-structured interviews or primarily qualitative methods for evaluating program effectiveness meaning they were excluded from our review, although these also found a reduction in participants' stigmatizing attitudes[58].

In sum, our main objective was to review effective workplace-based interventions for addressing mental health related stigma with a particular focus on SMEs. Unfortunately, our results did not entirely meet our expectations, as none of the reviewed interventions targeted SMEs specifically. Possible reasons behind this may be due to data protection reasons as limited data on the exact size and type of the organisations were noted. Most of the interventions were conducted in larger companies or public organizations, and therefore it is difficult to determine their feasibility in smaller enterprises with smaller numbers of employees and supervisors. However, we identified positive effects in studies where differently sized companies participated. Stigma-reduction in SME workplaces therefore remains unaddressed, although our review did add some new perspectives for smaller enterprises.

Our purpose to review interventions with appropriate methodology has produced rather positive results. The reviewed papers indicate that the included interventions produced for the most part significant reductions in stigmatizing attitudes for both employees and managers, and despite variation in methodology, common conclusions could be drawn.

Limitations

Notwithstanding the positive results of this review, several limitations should be mentioned. Only English language articles were included from five electronic databases, but we did not use occupational health databases for primary literature.

We have identified a clear dominance of interventions targeting higher educated white collar employers and employees, inhibiting the generalizability of effectiveness to less educated or blue-collar employees. In addition, all studies were conducted in either European countries, North-America, Australia or Japan therefore not representing experiences from other parts of the world, with larger parts of the populations with lower economic status. Only studies with quantitative measurement were included in this review, however studies with interview or focus group designs could provide important additional information. Similarly, we did exclude studies with no direct measure on stigma, however attitudes towards mentally ill patients and knowledge of mental health are important factors of stigmatizing behavior. Given the diverse study designs and outcome measures, it was not possible to conduct a meta-analysis.

Having based our review on quantitative studies we found that most programs were effective in changing stigmatizing attitudes and in some studies also were able to lead to behaviour change. However, this review does not provide a better understanding of the mechanisms that

For peer review only

lead to these changes. The knowledge about the effectiveness of the anti-stigma interventions presented in this review therfore should be supplemented with other reviews, including more or only qualitative studies, to investigate these aspects. Another important aspect of future studies can be the evaluation of which elements of interventions act on the level of individual and structural stigma separately. Again this also requires studies based on qualitative methodology.

Conclusions

A large proportion of the workforce could benefit from workplace-based interventions aimed at reducing mental health related stigma. Although we did not find interventions focusing specifically on SMEs, we can derive important findings from our review. Online anti-stigma interventions could have several benefits for smaller enterprises, they are shorter, and appear to have the same positive effects on stigmatizing attitudes as face-to-face interventions. These could be very important factors for professionals when trying to choose an intervention for their company.

Furthermore, investigations of the feasibility of these programs in smaller enterprises with less resources are needed, and more studies should go beyond measuring only attitudes.

Contributorship Statement

MDT, GYP, SI and EA conceived the idea for the study. MDT, GYP, SI, BA and CL planned the study design. MDT, SI and GYP carried out the search and quality assessments with input from BA and CL. MDT and GYP wrote the first draft of the report with input from BA, JCS, HR, GC, SS, NF, GQ, FT, VR, SM and ACP. All authors contributed to the interpretation of findings and critical revision of the manuscript. All authors approved the final version of the manuscript for submission, MDT submitted the manuscript.

Competing interests

The authors declare no competing interests.

Funding

This work was supported by the European Union's Horizon 2020 Research and Innovation Programme grant number 848137.

Data sharing statement

No additional data available.

Ethics approval

Ethical approval was given by the Hungarian Medical Research Council, reference number: IV/10156-3/2020/EKU.

References

[1] Flores EC, Fuhr DC, Bayer AM et al. Mental health impact of social capital interventions: a systematic review. *Soc Psychiatry Psychiatr Epidemiol* 2018;53(2): 107-119.

For peer review only

[2] Leka S, Jain A, Iavicoli S et al. An evaluation of the policy context on psychosocial risks and mental health in the workplace in the European Union: achievements, challenges, and the future. *BioMed research international*, *2015*.

[3] Johnston DA, Harvey SB, Glozier N et al. The relationship between depression symptoms, absenteeism and presenteeism. *J Affect Disord* 2019;256: 536-540.

[4] Bryan ML, Bryce AM, Roberts J. Presenteeism in the UK: Effects of physical and mental health on worker productivity. The Sheffield Economic Research Paper Series (SERPS) 2020; 2020005(2020005).

[5] Niedhammer I, Bertrais S, Witt K. Psychosocial work exposures and health outcomes: a meta-review of literature reviews with meta-analysis. S*cand J Work Environ Health* 2021;47(7):489–508.

[6] Bubonya M, Cobb-Clark DA, Wooden M. Mental health and productivity at work: Does what you do matter? *Labour Econ* 2017;46: 150-165.

[7] Brouwers EP. Social stigma is an underestimated contributing factor to unemployment in people with mental illness or mental health issues: position paper and future directions. *BMC Psychol* 2020;8(1):1-7.

[8] WHO, 2017: Depression and Other Common Mental Disorders: Global Health Estimates. Geneva: World Health Organization; 2017. Licence: CC BY-NC-SA 3.0 IGO.

[9] Drew JM, Martin S. A National Study of Police Mental Health in the USA: Stigma, Mental Health and Help-Seeking Behaviors. *J Police Crim Psych* 2021;36: 295–306.

[10] Link BG, Phelan JC. Conceptualizing stigma. *Annu Rev Sociol* 2001;27(1): 363-385.

[11] DeFreitas SC, Crone T, DeLeon M et al. Perceived and personal mental health stigma in Latino and African American college students. *Front Public Health* 2018;6: 49.

[12] Griffiths KM, Christensen H, Jorm AF et al. Effect of web-based depression literacy and cognitive–behavioural therapy interventions on stigmatising attitudes to depression: Randomised controlled trial. *Br J Psychiatry* 2004;185(4): 342-349.

[13] Arnaez JM, Krendl AC, McCormick BP et al. The association of depression stigma with barriers to seeking mental health care: A cross-sectional analysis. *J Ment Health* 2020;29(2):182-190.

[14] Fox AB, Smith BN, Vogt D. How and when does mental illness stigma impact treatment seeking? Longitudinal examination of relationships between anticipated and internalized stigma, symptom severity, and mental health service use. *Psychiatry Res* 2018;*268*: 15-20.

[15] Haugen PT, McCrillis AM, Smid GE et al. Mental health stigma and barriers to mental health care for first responders: A systematic review and meta-analysis. *J Psychiatr Res* 2017;94: 218-229.

For peer review only - http://bmjopen.bmj.com/site/about/guidelines.xhtml

[16] Couture SM, Penn D. Interpersonal contact and the stigma of mental illness: A review of the literature. *J Ment Health* 2003;12(3): 291–305.

[17] Krupa T, Kirsh B, Cockburn L, Gewurtz R. Understanding the stigma of mental illness in employment. *Work* 2009;33(4): 413-425.

[18] LaMontagne AD, Martin A, Page KM et al. Workplace mental health: Developing an integrated intervention approach. *BMC Psychiatry* 2014;14(1): 131.

[19] Pinheiro M, Ivandic I, Razzouk D. The economic impact of mental disorders and mental health problems in the workplace. In Mental Health Economics (pp. 415-430). Springer, Cham, 2017.

[20] McDaid D, King D, Parsonage M. In: Knapp M, McDaid D, Parsonage M (eds). Mental health promotion and prevention: the economic case. London: Department of Health, 2011:20-1.

[21] Thorpe K, Chénier L. Building mentally healthy workplaces: perspectives of Canadian workers and front-line managers. Ottawa: Conference Board of Canada, 2011

[22] European Commission (2022): https://ec.europa.eu/growth/smes/sme-definition_en. Downloaded: 15th February, 2022.

[23] Gayed A, Tan L, LaMontagne AD et al. A comparison of face-to-face and online training in improving managers' confidence to support the mental health of workers. *Internet Interv* 2019;18: 100258.

[24] Hanisch SE, Twomey CD, Szeto AC et al. The effectiveness of interventions targeting the stigma of mental illness at the workplace: a systematic review. *BMC Psychiatry 2016*;16(1): 1-11.

[25] Arensman E, O'Connor C, Leduc C et al. Mental Health Promotion and Intervention in Occupational Settings: Protocol for a Pilot Study of the MENTUPP Intervention. *Int J Environ Res Public Health* 2022;19(2): 947.

[26] Page MJ, Moher D, Bossuyt PM et al. PRISMA 2020 explanation and elaboration: Updated guidance and exemplars for reporting systematic reviews. *BMJ*, 2021; 372: n160.

[27] Ciliska D, Miccouci S, Dobbins M. Effective public health practice project. quality assessment tool for quantitative studies. Hamilton, On: Effective Public Health Practice Project 1998.

[28] Griffiths KM, Bennett K, Walker J et al. Effectiveness of MH-Guru, a brief online mental health program for the workplace: A randomised controlled trial. *Internet inter* 2016;6: 29-39.

[29] Hanisch SE, Birner UW, Oberhauser C et al. Development and evaluation of digital game-based training for managers to promote employee mental health and reduce mental illness stigma at work: Quasi-experimental study of program effectiveness. *JMIR Ment Health* 2017;4(3): e31.

For peer review only

[30] Shann C, Martin A, Chester A et al. Effectiveness and application of an online leadership intervention to promote mental health and reduce depression-related stigma in organizations. *J Occup Health Psychol* 2019;24(1): 20.

[31] Paterson H, Todorova GK, Noble K et al. Evaluation of Headtorch WORKS as a workplace intervention for improved support and understanding of co-workers with poor mental health and well-being. *Eur J Work Organ Psychol* 2021; 1-12.

[32] Griffiths KM, Christensen H, Jorm AF. Predictors of depression stigma. *BMC Psychiatry* 2008;*8*(1): 1-12.

[33] Griffiths KM, Batterham PJ, Barney, L et al. The Generalised Anxiety Stigma Scale (GASS): psychometric properties in a community sample. *BMC Psychiatry*, 2011;11(1): 1-9.

[34] Martin A. Individual and contextual correlates of managers' attitudes toward depressed employees. *Human Resource Management* 2010;49(4): 647-668.

[35] King M, Dinos S, Shaw J et al. The stigma scale: Development of a standardised measure of the stigma of mental illness. *Br J Psychiatry* 2007;190: 248–254.

[35] Szeto AC, Luong D, Dobson KS. Does labeling matter? An examination of attitudes and perceptions of labels for mental disorders. *Soc Psychiatry Psychiatr Epidemiol* 2013;48(4): 659-671.

[36] Tynan RJ, James C, Considine R et al. Feasibility and acceptability of strategies to address mental health and mental ill-health in the Australian coal mining industry. *Int J Ment Health Syst* 2018;12(1): 66.

[37] Eiroa-Orosa FJ, Lomascolo M, Tosas-Fernández A. Efficacy of an Intervention to Reduce Stigma Beliefs and Attitudes among Primary Care and Mental Health Professionals: Two Cluster Randomised-Controlled Trials. *Int J Environ Res Public Health* 2021;18(3): 1214.

[38] Hamann J, Mendel R, Reichhart et al. A "Mental-Health-at-the-Workplace" Educational Workshop Reduces Managers' Stigma Toward Depression. *J Nerv Ment Dis* 2016;204(1): 61-63.

[39] Moffitt J, Bostock J, Cave A. Promoting well-being and reducing stigma about mental health in the fire service. *J Public Ment Health* 2014;12(2): 103-113.

[40] Svensson B, Hansson L. Effectiveness of mental health first aid training in Sweden. A randomized controlled trial with a six-month and two-year follow-up. *PloS one* 2014;9(6): e100911.

[41] Dimoff JK. Mental health awareness training (MHAT): the development and evaluation of an intervention for leaders. *Int J Stress Manag* 2016;23(2): 167–189.

[42] Dobson KS, Markova V, Wen A et al. Effects of the Anti-stigma Workplace Intervention "Working Mind" in a Canadian Health-Care Setting: A Cluster-Randomized Trial of Immediate Versus Delayed Implementation. *Can J Psychiatry* 2021;66(5): 495-502.

[43] Gast M, Lehmann J, Schwarz E et al. A Single-Day Training for Managers Reduces Cognitive Stigma Regarding Mental Health Problems: A Randomized Trial. *Int J of Environ Res Public Health* 2022;19(7), 4139.

[44] Kassam A, Papish A, Modgill G et al. The development and psychometric properties of a new scale to measure mental illness related stigma by health care providers: the Opening Minds Scale for Health Care Providers (OMS-HC). *BMC Psychiatry* 2012;12(1): 1-12.

[45] Bond KS, Cottrill, FA, Mackinnon, A et al. Effects of the Mental Health First Aid for the suicidal person course on beliefs about suicide, stigmatising attitudes, confidence to help, and intended and actual helping actions: an evaluation. *Int J Ment Health Syst* 2021;15(1):1-12.

[46] Kubo H, Urata H, Katsuki R et al. Development of MHFA-based 2-h educational program for early intervention in depression among office workers: A single-arm pilot trial. *PloS One* 2018;13(12): e0208114.

[47] Kristman VL, Lowey J, Fraser L et al. A multi-faceted community intervention is associated with knowledge and standards of workplace mental health: the Superior Mental Wellness@ Work study. *BMC Public Health* 2019;19(1): 638.

[48] Quinn N, Smith M, Fleming S et al. Self and others: the differential impact of an anti-stigma programme. *Stigma Res Action* 2011;1(1): 36-43.

[49] Braunholtz S, Davidson S, King S. Well? What do you think? The second national Scottish survey of public attitudes to mental health, mental well-being and mental health problems. Edinburgh: Scottish Executive, 2004.

[50] Stelnicki AM, Jamshidi L, Fletcher AJ, Carleton RN. Evaluation of before Operational Stress: A Program to Support Mental Health and Proactive Psychological Protection in Public Safety Personnel. *Frontiers in psychology*. 2021:3218.

[51] Dobson KS, Szeto A, Knaak S. The Working Mind: A meta-analysis of a workplace mental health and stigma reduction program. *Can J Psychiatry* 2019;64(1): 39S-47S.

[52] Szeto A, Dobson KS, Knaak S. The road to mental readiness for first responders: a meta-analysis of program outcomes. *Can J Psychiatry* 2019;64(1): 18S-29S.

[53] Kelly BJ, Stain HJ, Coleman C et al. Mental health and well-being within rural communities: the Australian rural mental health study. *Aust J Rural Health* 2010;18:16–24.

[54] Moll SE, Patten S, Stuart H et al. Beyond silence: a randomized, parallel-group trial exploring the impact of workplace mental health literacy training with healthcare employees. *Can J Psychiatry* 2018;63(12): 826-833.

[55] Reavley NJ, Morgan AJ, Fischer JA et al. Effectiveness of eLearning and blended modes of delivery of Mental Health First Aid training in the workplace: randomised controlled trial. *BMC Psychiatry* 2018;18(1): 1-14.

For peer review only

[56] Reavley NJ, Morgan AJ, Fischer JA et al. Longer-term effectiveness of eLearning and blended delivery of Mental Health First Aid training in the workplace: 2-Year follow-up of a randomised controlled trial. *Internet interventions*. 2021;(1)25:100434.

[57] Lam LT, Lam MK, Reddy P, Wong P. Efficacy of a workplace intervention program with Web-based online and offline modalities for improving workers' mental health. *Frontiers in Psychiatry*. 2022; 31:975.

[58] Ross V, Caton N, Gullestrup J et al. Understanding the barriers and pathways to male help-seeking and help-offering: a mixed methods study of the impact of the Mates in Construction Program. *Int J Environ Res Public Health* 2019;16(16): 2979.

For peer review only

PRISMA 2020 flow diagram for new systematic reviews which included searches of databases and registers only

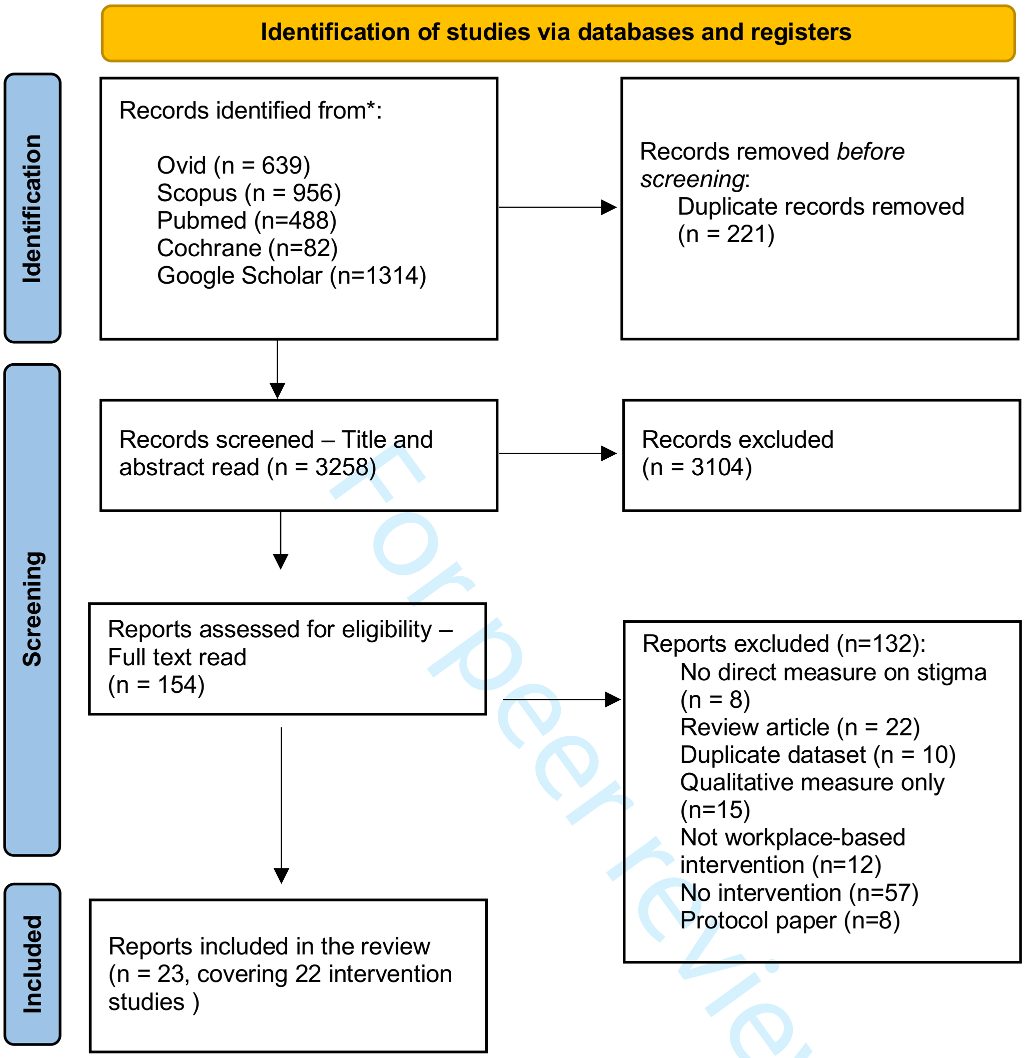

For peer review only

From: Page MJ, McKenzie JE, Bossuyt PM, Boutron I, Hoffmann TC, Mulrow CD, et al. The PRISMA 2020 statement: an updated guideline for reporting systematic reviews. BMJ 2021;372:n71. doi: 10.1136/bmj.n71

For more information, visit: http://www.prisma-statement.org/

Appendix 1:

The following TI/AB keywords were used:

depress* OR suic* OR anx* OR self-harm OR "mental health" OR discrimination OR exclusion
AND
occupation* or workplace or SME OR job OR "small-sized enterprise*" OR "medium-sized enterprise*" OR "small enterprise*" OR "medium enterprise*" OR "small-sized compan*" OR "medium-sized compan*" OR "small compan*" OR "medium compan*" OR "small-sized business*" OR "medium-sized business*" OR "small business*" OR "medium business*" OR "small-sized organization*" OR "small-sized organisation*" OR "medium-sized organization*" OR "medium-sized organisation*" OR "small organization*" OR "small organisation*" OR "medium organization*" OR "medium organisation*")
AND
anti-stigma OR stigma
AND
reduced OR promot* OR program* OR campaign OR improve* OR intervention OR educat* OR seminar* OR workshop* OR course

For peer review only

Supplementary table 1. Overview of study characteristics

First author/ year	Study Design	Population at baseline and follow up	Gender at baseline	Target group	Sector/ Size of organization	Intervention	Intervention Intensity	Country
**Bond et al, 2021**	longitudinal cohort study	284 pre<br>98 post	212 female<br>72 male	support services, police, educators and general community networks	public sector	Mental Health First Aid for the Suicidal Person course	4-hours course	Australia
**Dimoff 2016**	controlled study active vs wait list	183 pre<br>142 post	77 female<br>65 male	Leaders in telecommunication companies	large company, private sector	Mental health awareness training	3 hours training	Canada
**Dobson et al, 2021**	cluster-randomized trial	123 pre<br>101 post	115 female<br>8 male	office workers<br><br>kitchen and maintenance staff	large company, public sector	The Working Mind program:<br>• trained facilitators,<br>• workshop manuals,<br>• contact-based videos<br>• discussion exercises,<br>• personal goal setting.<br>Participants allocated to immadiate or delayed intervention groups.	4-hours group program	Canada
**Dobson et al., 2019**	open trial methodology	1292 pre<br>1155 post	male 419<br>female 719	government, education, health, energy supervisors and frontline staff	public sector	The Working Mind Program<br>• trained facilitators,<br>• workshop manuals,<br>• contact-based videos<br>• discussion exercises,<br>• personal goal setting.<br>"train-the- trainer" model	Two versions:<br>4-hour group program for frontline workers<br><br>8-hour program for managers	Canada
**Eiroa-Orosa et al, 2021**	cluster randomized-	371 pre<br>260 post	314 female<br>57 male	primary health and mental health care professionals	public sector	awareness-raising intervention<br><br>The Targeted, Local, Credible, Continuous Contact (TLC3)	**4 workshops**<br>1. Training: pedagogy and contact (face-to face +video) – 4 hours	Spain

	controlled trial			administrative officers, general practitioners, odontologists, nurses, psychiatrists, psycholhologists, and social workers.		methodology adapted to the Catalan healthcare context	2. self-diagnosis and prioritization – 4 hours<br>3. self-organized activities<br>4. follow-up session	
**Griffith et al 2016**	Randomised controlled trial	507 pre<br>386 post	MH-guru: male: 29%, female: 70%;	multi-departmental government workplace	public sector	online depression and anxiety educational workplace induction program ("Mental Health Guru"): two modules: depression and General anxiety disorder	1 hour long online depression and anxiety educational program (1 module/week, 30min/module)	Australia
**Hamann et al., 2016**	Longitudinal cohort study	580 pre	210 women<br>370 men	Leaders, members of the workers' council, workers in HR department	not specified companies (n=30)	"Mental-health-at-the-workplace" educational workshop	1-1,5 days training	Germany
**Hanisch et al., 2017**	Longitudinal cohort study	48 pre	92% male, 8% female	Leaders	private sector – large enterprise	"Leadership Training in Mental Health Promotion" (LMHP), a digital game-based training program for leaders which is combining games and simulations in a virtual environment.	1.5- 2 hours long single session	UK
**Kristman et al., 2019**	quasi-experimental	89 pre<br>61 post	59 male<br>24 female	Leaders in HR, occupational health and safety management	public and private sector, different size companies	Multi-faceted:<br>1. "Standard to Action" training program designed to help employers implement the Standard in	2 years	Canada

						their workplaces – 6 sessions;<br>2. Education sessions: MH First Aid sessions;<br>3. Social marketing campaign including a photovoice exhibit.		
**Kubo et al, 2018**	Single arm pilot trial	91 pre<br>83 post	male 77%<br>female 23%	office workers	no specific informatio n	"Mental Health First Aid" (MHFA) training program modified for workplace settings.	2-hour training course	Japan
**Moffitt et al, 2014**	random allocation design	106 pre<br>89 post	N/A	fire service line managers	public sector	Participants randomly assigned to:<br>• Looking after Wellbeing at Work" (LWW)<br>• Mental Health First Aid (MHFA)<br>• leaflet session (LS).	LWW- 2days<br><br>MHFA – 12 hours<br><br>LS- 1 hour	UK
**Moll et al, 2018**	randomise d, parallel-group trial	192 pre<br>167 post<br>150 by 6 mo follow up	female 88.5%<br>male 11.5%	Healthcare workers	public sector	"Beyond Silence" (Beyond Silence program includes a contact-based educational approach)<br><br>Mental Health First Aid training	comprising 6 in-person, 2-h sessions + 5 online sessions co-led by employees who personally experienced mental health issues<br>standardised 2-days training program	Canada

**Paterson et al, 2021**		134 pre<br>57 post	not specified	not specified	public and private, different size companies	Headtorch WORKS - mental health and well-being intervention	3 online episodes + discussion group<br>6 hours<br>original filmed drama and specialist documentary	UK
**Quinn et al, 2011**		101 pre<br>87 post	77% male<br>23% female	housing association and telecommunication workers	public and private sector	Training course on mental health awareness	One day training (6 hours each)<br><br>combination of service user narratives, experiential group learning, and didactic teaching approaches.	Scotland
**Reavley et al., 2018**	Randomized controlled trial	608 pre<br>289 post	449 female<br>159 male	public servants	public sector	Participants randomized to<br><br>-eLearning MHFA,<br><br>-blended MHFA<br><br>-PFA eLearning	- 6-hour eLearning MHFA online course<br>- 6-hour eLearning MHFA plus 4-hour face-to-face session<br>- 4-hour eLearning PFA online course	Australia
**Shann et al., 2018**	Randomized controlled trial	311 pre<br>196 post	148 male<br>163 female	Leaders	public<br>private<br>non- profit<br>other sectors 1%	"Beyondblue" online materials for leaders:<br>Main focus on depression:<br>-written information,<br>-video clips of organizational leaders speaking about mental health in the workplace,<br>-interactive exercises in which participants can calculate the cost of untreated depression and the	30 – 45 min	Australia

						specific risk factors in their organization.		
**Svensson and Hansson, 2014**	Randomized controlled trial	416 pre<br>277 post	151 female<br>48 male	Not specified employees	public sector	Mental Health First Aid training	12 hours course, spread over two days	Sweden
**Szeto et al. 2019**	non-randomized quasi-experimental	5598 pre<br>4649 post<br><br>Frontline staff 75.8% (3,449)<br>Supervisory staff 26.4% (1,210)	male 55.9%<br>female 44.1%	Corrections 9.0% (418)<br>Emergency Services (9-1-1) 3.9% (192)<br>Fire Services 17.7% (821)<br>Police Services 56.5% (2,623)<br>Paramedics 13.0% (605)	public sector	"Road to Mental Readiness for First Responders" program (R2MR)<br>**3 main components**:<br>stigma reduction through video contact-based education,<br>the Mental Health Continuum Model, and<br>"Big 4" coping and resilience skills.<br>Additional skills for supervisors.	4-hour program for employees<br><br>8-hour program for supervisors	Canada
**Tynan et al. 2018**	Non-Randomised controlled trial	1275 pre<br>1163 post<br><br>Supervisor:<br>117 pre<br>114 post	1014 male;<br>135 female;<br><br>Supervisor training:<br>92 male;<br>10 female;<br>12 not specifed.	Manager<br>Professional<br>Trades worker<br>Machinery operator Admin or other	private sector, medium and large	"Working Well Mental health Program":<br><br>peer-based, multi-component mental health and suicide prevention program<br><br>supervisor training	- 1 hour 'general awareness training' (GAT),<br>- 4 hours of 'gate-keeper training',<br>-2-day 'Applied Suicide Intervention Skills Training' (ASIST) for key workers.	Australia

Supplementary table 2. Study findings

**Results from Randomized Controlled Trials**				
**First author/year**	**Outcome measure on stigma**	**Evaluation timepoints**	**Main findings**	
Dimoff 2016	Depression Stigma personal Scale (DSS)	pre training<br>post training<br><br>2 months follow up	Significant improvements in stigmatizing attitudes were also observed for the intervention group from T1 (M = 2.98, SD = 0.39) to T2 (M =3.25, SD = 0.37; t (87) = -5.60, p < .001) and from T1 to T3 (M = 3.20, SD = 0.42; t (87) = -4.06, p < .001).<br>No significant improvements in attitude were observed for the intervention group between T2 and T3.	
Dobson et al, 2021	Opening Minds Scale for Workplace Attitudes (OMS-WA)	pre, post training<br>3 months follow up	Stigma scores on the OMS-WA revealed a significant time effect, F(2/154) = 16.33, P < 0.001. There was also a significant group effect, F(1/76) = 16.23, P <0.001, but the interaction effect was not statistically significant, F(2/154) = 1.02, P = 0.362.<br>Pairwise comparison analyses revealed a significant pre- to postreduction in stigma for both the immediate, t(154) = 3.22, P = 0.004, and the delayed group, t(154) = 4.12, P < 0.001.<br>Significant reduction in stigma from pre- to posttest, which was maintained to the time of the follow-up assessment.	
Eiora-Orosa et al, 2021	Opening Minds Scale for Health Care Providers (OMS-HC)<br><br>Beliefs and Attitudes towards Mental Health Service users' rights	pre,<br>1 month follow-up<br>3 months follow up	At baseline statistically significant difference between the intervention and control groups in the total score of the OMS-HC scale ($t = 2.138$, $p < 0.05$)<br><br>Statistically significant decreases were seen between baseline and first follow-up for the OMS-HC total score ($t = 2.813$, $p < 0.01$)<br><br>The general linear models showed a statistically significant drop between the first observation and the second for the OMS-HC disclosure scores with statistically significant effects ($F = 26.881$, $p < 0.001$)<br><br>Reductions in both PC and MH professionals' stigmatising beliefs and attitudes were found in the 1-month follow-up, although a 'rebound effect' at the 3-month follow up was detected.	

For peer review only

Griffith et al. 2016	depression and anxiety personal stigma scale (DSS-personal) (GASS-personal)	baseline,<br>1 week post-intervention<br>6-month follow-up	MH-Guru group showed significantly greater ↓in depression and anxiety personal stigma. Between group effect sizes in stigma for depression were − 0.56 and − 0.47 at post-test and 6-months respectively and − 0.42 at both time points for anxiety (p<.001)<br><br>DSS (Mean, SD)<br>MH-Guru: before: 7.1 (4.9) after: 3.9 (3.8) follow up 4.2 (3.8)<br>Control: Before: 7.3 (5.2) after: 6.8 (5.0) follow up: 6.6 (5.2)<br>F (2, 294.1)=2.5 *P*<.001<br><br>GASS (mean, SD)<br>MH-guru: before: 5.1 (5.1) after: 2.5 (3.9) follow up: 5.1 (0.48)<br>control: before: 4.9 (5.6) after: 5.0 (5.3) follow up: 4.9 (0.34)<br>F (2, 286.1)=19.8 p <.001	
Moffitt, 2014	locally developed "Mental Health Stigma Questionnaire"	pre, post intervention	The LWW and MHFA courses were associated with statistically significant improvements in stigma on mental health.<br>The comparisons showed no significant difference at Time 2 between the LWW and MHFA groups on stigma scale (z= 0.57, p = 0.57, r = 0.07).	
Reavley et al, 2018	Personal Stigma Scale (PSS)	Pre, post training	Those in the blended and eLearning MHFA groups were significantly more likely to show reduced stigma towards people with depression and PTSD than those in the PFA eLearning group.<br>No significant differences between the MHFA eLearning and blended courses.	
Shann et al. 2018.	Managerial Stigma Toward Employee Depression Scale<br>- Affective Stigma Subscale,<br>- Behavioral Stigma Subscale,	pre – post intervention<br>6 month follow up	Significant reductions in behavioral and affective depression-related stigma scores among leaders who completed the intervention, same reduction at 6 months.<br><br>One-way multivariate analysis of covariance showed a statistically significant difference in survey stigma between experimental and control groups, V =.09, F(3, 189)= 6.26, p <.001.<br>Follow-up univariate analyses of variance showed that at posttest, affective stigma was significantly different between groups, F(1, 191) = 14.55, p <.001.	

For peer review only

	- Cognitive Stigma Subscale		The experimental group had lower affective stigma scores (M= 9.42, SEM= .24) at postsurvey compared with the control group (M= 10.51, SEM= .16).	
Svensson and Hansson, 2014	vignette version of the Depression Personal and Perceived Stigma scale (DSS)	pre<br>6 months and<br>2 years follow up	Significant reduction in depression personal stigma after 6 months follow up. Intervention group: pre: 35,8 (5,2) post: 36,3 (4,8). Control group: pre: 36,4 (4.5) post: 35.4 (5.3). F=6,3 p<.05, effect size:0,29.<br><br>The training after two years still have a notable impact on the awareness of mental health and its treatment.	
**Results from quasi-experimental or pre-post design studies**				
Bond et al., 2021	9 statements designed to measure stigmatising attitudes based on Depression Stigma Scale (DSS) – suicide vignette	pre, post training,<br>6 month follow-up	Reductions in scores on „weak not sick" item after the course and at follow-up (t(275.6)=8.89, p<.0001 and t(132.7)=2.66, p<.0001. Changes in means of „Dangerous/unpredictable"item from pre-course were signifcant both postcourse and at follow-up (t(267.0)=11.74, p<.0001 and t(125.5)=3.81, p=0.0002, respectively).	
Dobson et al., 2019	- Stigma towards mental health problems (OMS-WA)	pre program<br>post program<br>3-month follow-up period	The results of the mixed-model analysis revealed statistically significant ↓ in stigma for the total scale, coefficient = .167, SE = .08, z = 20.72, P < 0.001, and all subscales (all Ps < 0.001).<br>The mixed-model analysis for the pre- to post- change on the resiliency skills scale revealed statistically significant improvement at the 95% level of confidence (P < 0.001).	
Hamann et al., 2016	Depression Stigma Scale (DSS)	Pre, post training	significant ↓in personal stigma (mean [SD], 15.5 [3.8]; paired t-test: t = 27.6, p < 0.001)	
Hanisch et al, 2017	-Stigma towards mental health problems (OMS-WA)	pre, post-training, 3-month follow-up	Positive changes on attitudes toward people with mental health problems (*P*<.01).	
Kristman et al., 2019	-Perceived mental health stigma on the workplace	presurvey<br>postsurvey - 2 yrs	Intervention group - pre: mean (SD)= 1.84 (0.74) post: 1.71 (0.64) MD: -0.13, 0.11)	

For peer review only

	Questions derived from Workplace Mental Health in Canada survey.		Significant difference in perceived mental health stigma btw intervention and non-intervention group:<br><br>Intervention group: mean (SD): 1.52 (0.57) vs. Non-intervention group: 2.00 (0.63), MD: -0.48, 95% CI	
Kubo et al. 2018	-Stigma towards mental health problems:<br>Link's Devaluation-Discrimination Scale	pre-program, post-program,<br>1 month follow up	↓ after the program (before: mean (SD)=28.29 (4.9), after: mean (SD) 26.11 (5.36) p=0.003),<br>no difference 1 month after the program. mean (SD): 27.26 (5.78)	
Moll et al. 2018	Stigma towards mental health problems – health care (OMS-HC)	presurvey<br>Postsurvey<br>3-mo assessment, 6-mo follow-up	Stigmatized beliefs significantly ↓ in both programs.<br><br>In the stigma analysis, no interactions for treatment arm by time were observed at 3 mo (beta = 0.21, z = 0.22, P = 0.83); although, a possible trend for superior outcomes for Beyond Silence was seen at 6 mo (beta = 1.72, z = 1.7, P = 0.089). To explore whether the anti-stigma effects of Beyond Silence might be more persistent than those of MHFA, a model describing changes from 3 to 6 mo was fit, revealing a significant treatment by time interaction (beta = 1.89, z = 2.09, P 1⁄4 0.037).	
Paterson et al, 2021	Adopted version of King's stigma scale[1]	pre, post intervention	There was no significant difference in the pre/post-intervention change in stigma score between the experimental and control groups.	
Quinn et al, 2011	questions gathered from the Scottish Public Attitudes Survey[2]	pre, post intervention	Attendance at the workshop reduced the level of stigmatizing attitudes for both first (t = 11.939, df = 86, p < 0.0005) and third (t = 3.535, df = 86, p = 0.001) person views. The workshop was associated with a more marked reduction in stigmatizing attitudes	

[1] King, M., Dinos, S., Shaw, J., Watson, R., Stevens, S., Passetti, F., . . . Serfaty, M. (2007). The stigma scale: Development of a standardised measure of the stigma of mental illness. British Journal of Psychiatry, 190(MAR.), 248–254

[2] Braunholtz, S., Davidson, S., & King, S. (2004). Well? What do you think? The second national Scottish survey of public attitudes to mental health, mental well-being and mental health problems. Edinburgh: Scottish Executive.

			expressed by first compared with third person views.	
Szeto et al. 2019	Stigma towards mental health problems (OMS-WA)	pre-program, post-program, 3 month follow up	↓in stigma were observed for the total scale and all subscales. before: 1.97 (SD: 0.47). After: 1.85 (SD: 0.49) coeff: 0.123 SE: 0.008 z: 15.87 p<0.001 Reductions in stigma were maintained until the final follow-up for the total scale. coeff: - 0.002 SE: 0.012 z: - 0.13 p=0.899	
Tynan, 2018	-Mental health stigma, measured by *a perceived stigma* scale[3]	pre-test post-test 10 months follow up	Trend towards a decrease in stigma across both control and intervention sites, however the effect of time or treatment was not signifcant (p > 0.01)	

Key. DSS: Depression Stigma Scale. GASS: The Generalised Anxiety Stigma scale, OMS-WA: Opening Minds Scale for Workplace Attitudes, OMS-HC: Opening Minds Scale for Health Care Providers, PSS: Personal Stigma Scale.

[3] Kelly BJ, Stain HJ, Coleman C, Perkins D, Fragar L, Fuller J, Lewin TJ, Lyle D, Carr VJ, Wilson JM, Beard JR. Mental health and well-being within rural communities: the Australian rural mental health study. *Aust J Rural Health*. 2010;18:16–24.

For peer review only

Supplementary table 3: Quality assessment of the selected studies[1]

Quality of the selected studies		
WEAK	MODERATE	STRONG
Bond et al, 2021	Dimoff et al, 2016	Moll et al, 2018
Kristman et al, 2019	Dobson et al, 2019	Svensson and Hansson, 2014
Kubo et al, 2018	Dobson et al, 2021	
Paterson et al, 2021	Eirosa-Orosa et al, 2021	
Quinn et al, 2011	Griffith et al, 2016	
	Hamann et al, 2016	
	Hanisch et al, 2017	
	Moffitt et al, 2014	
	Reavley et al, 2018	
	Shann et al, 2018	
	Szető et al, 2019	
	Tynan et al, 2018	

1: Based on Quality Assessment Tool for Quantitative Studies (QATQS) scale (Ciliska et al, 1998)

For peer review only

Supplementary Table 4: Quality Assessment of the included studies, based on the QATQS

First Author	Selection Bias	Design	Confounders	Blinding	Data Collection Method	Withdrawals and Drop-out	Global Rating
**Bond et al, 2021**	Strong	Moderate	Weak	Moderate	Strong	Weak	**Weak**
**Dimoff et al, 2016**	Weak	Strong	Strong	Strong	Strong	Moderate	**Moderate**
**Dobson et al, 2019**	Moderate	Moderate	Weak	Moderate	Strong	Strong	**Moderate**
**Dobson et al, 2021**	Moderate	Moderate	Weak	Strong	Strong	Strong	**Moderate**
**Eiroa-Orosa et al, 2021**	Moderate	Strong	Weak	Strong	Strong	Moderate	**Moderate**
**Griffith et al, 2016**	Weak	Strong	Strong	Strong	Strong	Moderate	**Moderate**
**Hamann et al, 2016**	Moderate	Moderate	Weak	Moderate	Strong	Strong	**Moderate**
**Hanisch et al, 2017**	Strong	Moderate	Weak	Moderate	Strong	Strong	**Moderate**
**Kristman et al, 2019**	Weak	Moderate	Weak	Moderate	Weak	Moderate	**Weak**
**Kubo et al, 2018**	Strong	Moderate	Weak	Weak	Strong	Strong	**Weak**
**Moffitt et al, 2014**	Moderate	Strong	Strong	Strong	Weak	Strong	**Moderate**
**Moll et al, 2018**	Moderate	Strong	Strong	Strong	Strong	Moderate	**Strong**
**Paterson et al, 2021**	Weak	Moderate	Weak	Strong	Strong	Weak	**Weak**
**Quinn et al, 2011**	Moderate	Moderate	Weak	Weak	Moderate	Strong	**Weak**
**Reavley et al, 2018**	Strong	Strong	Strong	Strong	Strong	Weak	**Moderate**
**Shann et al, 2018**	Weak	Strong	Strong	Strong	Moderate	Moderate	**Moderate**
**Svensson and Hansson, 2014**	Moderate	Strong	Strong	Strong	Strong	Moderate	**Strong**
**Szető et al, 2019**	Strong	Moderate	Weak	Moderate	Strong	Strong	**Moderate**

For peer review only

**Tynan et al, 2018**	Strong	Moderate	Strong	Strong	Weak	Moderate	**Moderate**

For peer review only

PRISMA 2020 Main Checklist

Topic	No.	Item	Location where item is reported
**TITLE**			
**Title**	1	Identify the report as a systematic review.	See Title pp 3.
**ABSTRACT**			
**Abstract**	2	See the PRISMA 2020 for Abstracts checklist	pp4
**INTRODUCTION**			
**Rationale**	3	Describe the rationale for the review in the context of existing knowledge.	See Introduction pp5
**Objectives**	4	Provide an explicit statement of the objective(s) or question(s) the review addresses.	See Introduction pp5
**METHODS**			
**Eligibility criteria**	5	Specify the inclusion and exclusion criteria for the review and how studies were grouped for the syntheses.	See study selection section pp6
**Information sources**	6	Specify all databases, registers, websites, organisations, reference lists and other sources searched or consulted to identify studies. Specify the date when each source was last searched or consulted.	See included studies section pp7
**Search strategy**	7	Present the full search strategies for all databases, registers and websites, including any filters and limits used.	See appendix 1 and search strategy section pp7 and pp37
**Selection process**	8	Specify the methods used to decide whether a study met the inclusion criteria of the review, including how many reviewers screened each record and each report retrieved, whether they worked independently, and if applicable, details of automation tools used in the process.	See study selection section pp6
**Data collection process**	9	Specify the methods used to collect data from reports, including how many reviewers collected data from each report, whether they worked independently, any processes for obtaining or confirming data from study investigators, and if applicable, details of automation tools used in the process.	See review procedure section pp6

For peer review only - http://bmjopen.bmj.com/site/about/guidelines.xhtml

Topic	No.	Item	Location where item is reported
**Data items**	10a	List and define all outcomes for which data were sought. Specify whether all results that were compatible with each outcome domain in each study were sought (e.g. for all measures, time points, analyses), and if not, the methods used to decide which results to collect.	See data extraction section pp 7
	10b	List and define all other variables for which data were sought (e.g. participant and intervention characteristics, funding sources). Describe any assumptions made about any missing or unclear information.	See data extraction section pp 7
**Study risk of bias assessment**	11	Specify the methods used to assess risk of bias in the included studies, including details of the tool(s) used, how many reviewers assessed each study and whether they worked independently, and if applicable, details of automation tools used in the process.	Supplementary Table 3. pp 29
**Effect measures**	12	Specify for each outcome the effect measure(s) (e.g. risk ratio, mean difference) used in the synthesis or presentation of results.	n/a
**Synthesis methods**	13a	Describe the processes used to decide which studies were eligible for each synthesis (e.g. tabulating the study intervention characteristics and comparing against the planned groups for each synthesis (item 5)).	n/a
	13b	Describe any methods required to prepare the data for presentation or synthesis, such as handling of missing summary statistics, or data conversions.	n/a
	13c	Describe any methods used to tabulate or visually display results of individual studies and syntheses.	n/a
	13d	Describe any methods used to synthesize results and provide a rationale for the choice(s). If meta-analysis was performed, describe the model(s), method(s) to identify the presence and extent of statistical heterogeneity, and software package(s) used.	n/a
	13e	Describe any methods used to explore possible causes of heterogeneity among study results (e.g. subgroup analysis, meta-regression).	n/a
	13f	Describe any sensitivity analyses conducted to assess robustness of the synthesized results.	n/a

For peer review only - http://bmjopen.bmj.com/site/about/guidelines.xhtml

Topic	No.	Item	Location where item is reported
**Reporting bias assessment**	14	Describe any methods used to assess risk of bias due to missing results in a synthesis (arising from reporting biases).	See Table 4. pp 31
**Certainty assessment**	15	Describe any methods used to assess certainty (or confidence) in the body of evidence for an outcome.	n/a
**RESULTS**			
**Study selection**	16a	Describe the results of the search and selection process, from the number of records identified in the search to the number of studies included in the review, ideally using a flow diagram.	See Result section pp8
	16b	Cite studies that might appear to meet the inclusion criteria, but which were excluded, and explain why they were excluded.	See study selection section pp6
**Study characteristics**	17	Cite each included study and present its characteristics.	Supplementary Table 1 and 2. pp 19 and 24.
**Risk of bias in studies**	18	Present assessments of risk of bias for each included study.	Supplementary Table 4. pp 31.
**Results of individual studies**	19	For all outcomes, present, for each study: (a) summary statistics for each group (where appropriate) and (b) an effect estimate and its precision (e.g. confidence/credible interval), ideally using structured tables or plots.	Supplementary Table 2. pp 24.
**Results of syntheses**	20a	For each synthesis, briefly summarise the characteristics and risk of bias among contributing studies.	n/a
	20b	Present results of all statistical syntheses conducted. If meta-analysis was done, present for each the summary estimate and its precision (e.g. confidence/credible interval) and measures of statistical heterogeneity. If comparing groups, describe the direction of the effect.	n/a
	20c	Present results of all investigations of possible causes of heterogeneity among study results.	n/a
	20d	Present results of all sensitivity analyses conducted to assess the robustness of the synthesized results.	n/a

For peer review only - http://bmjopen.bmj.com/site/about/guidelines.xhtml

Topic	No.	Item	Location where item is reported
**Reporting biases**	21	Present assessments of risk of bias due to missing results (arising from reporting biases) for each synthesis assessed.	n/a
**Certainty of evidence**	22	Present assessments of certainty (or confidence) in the body of evidence for each outcome assessed.	Supplementary Table 2. pp 24.
**DISCUSSION**			
**Discussion**	23a	Provide a general interpretation of the results in the context of other evidence.	See Discussion section pp 11.
	23b	Discuss any limitations of the evidence included in the review.	See limitation section pp 13
	23c	Discuss any limitations of the review processes used.	See limitation section pp 13
	23d	Discuss implications of the results for practice, policy, and future research.	See Conclusions section pp13
**OTHER INFORMATION**			
**Registration and protocol**	24a	Provide registration information for the review, including register name and registration number, or state that the review was not registered.	See data extraction section pp 7
	24b	Indicate where the review protocol can be accessed, or state that a protocol was not prepared.	See review procedure section pp 6
	24c	Describe and explain any amendments to information provided at registration or in the protocol.	n/a
**Support**	25	Describe sources of financial or non-financial support for the review, and the role of the funders or sponsors in the review.	See Funding section pp 14

For peer review only - http://bmjopen.bmj.com/site/about/guidelines.xhtml

Topic	No.	Item	Location where item is reported
**Competing interests**	26	Declare any competing interests of review authors.	See Competing interest interest section<br>pp 14
**Availability of data, code and other materials**	27	Report which of the following are publicly available and where they can be found: template data collection forms; data extracted from included studies; data used for all analyses; analytic code; any other materials used in the review.	n/A

For peer review only

PRIMSA Abstract Checklist

Topic	No.	Item	Reported?
**TITLE**			
**Title**	1	Identify the report as a systematic review.	Yes
**BACKGROUND**			
**Objectives**	2	Provide an explicit statement of the main objective(s) or question(s) the review addresses.	Yes
**METHODS**			
**Eligibility criteria**	3	Specify the inclusion and exclusion criteria for the review.	Yes
**Information sources**	4	Specify the information sources (e.g. databases, registers) used to identify studies and the date when each was last searched.	Yes
**Risk of bias**	5	Specify the methods used to assess risk of bias in the included studies.	Yes
**Synthesis of results**	6	Specify the methods used to present and synthesize results.	Yes
**RESULTS**			
**Included studies**	7	Give the total number of included studies and participants and summarise relevant characteristics of studies.	Yes
**Synthesis of results**	8	Present results for main outcomes, preferably indicating the number of included studies and participants for each. If meta-analysis was done, report the summary estimate and confidence/credible interval. If comparing groups, indicate the direction of the effect (i.e. which group is favoured).	Yes
**DISCUSSION**			
**Limitations of evidence**	9	Provide a brief summary of the limitations of the evidence included in the review (e.g. study risk of bias, inconsistency and imprecision).	Yes
**Interpretation**	10	Provide a general interpretation of the results and important implications.	Yes
**OTHER**			
**Funding**	11	Specify the primary source of funding for the review.	Yes
**Registration**	12	Provide the register name and registration number.	Yes

For peer review only - http://bmjopen.bmj.com/site/about/guidelines.xhtml

From: Page MJ, McKenzie JE, Bossuyt PM, Boutron I, Hoffmann TC, Mulrow CD, et al. The PRISMA 2020 statement: an updated guideline for reporting systematic reviews. MetaArXiv. 2020, September 14. DOI: 10.31222/osf.io/v7gm2. For more information, visit: www.prisma-statement.org

For peer review only

For peer review only - http://bmjopen.bmj.com/site/about/guidelines.xhtml
